# Reduced clinical severity during 2022 Shanghai Spring epidemic of SARS-CoV-2 omicron BA.2 variant infection—an integrated account of virus pathogenicity and vaccination effectiveness

**DOI:** 10.1093/nsr/nwae011

**Published:** 2024-01-15

**Authors:** Xingyue Wu, Yao Chen, Kangli Cao, Yao Shen, Xueling Wu, Yilin Yang, Zhongshu Kuang, Qingrun Li, Zhenzhen Lu, Yichen Jia, Mian Shao, Guorong Gu, Xiangwei Wang, Ye Yao, Ying Wang, Shaodie Chen, Zhigao Yu, Wei Wei, Longfei Ding, Lulu Lan, Tianwen Gu, Xiangyu Long, Jian Sun, Lingyu Xing, Jiayuan Shen, Yi Han, Yue Luo, Sucheng Mu, Mengna Lin, Xiaoyan Zhang, Rong Zeng, Jianqing Xu, Guoping Zhao, Lihong Huang, Zhenju Song

**Affiliations:** Department of Emergency Medicine, Clinical Center for Bio-Therapy, Department of Biostatistics, and Department of Urology, Shanghai Key Laboratory of Lung Inflammation and Injury, Zhongshan Hospital, Fudan University, China; Department of Emergency Medicine, Clinical Center for Bio-Therapy, Department of Biostatistics, and Department of Urology, Shanghai Key Laboratory of Lung Inflammation and Injury, Zhongshan Hospital, Fudan University, China; Department of Emergency Medicine, Clinical Center for Bio-Therapy, Department of Biostatistics, and Department of Urology, Shanghai Key Laboratory of Lung Inflammation and Injury, Zhongshan Hospital, Fudan University, China; Department of Respiratory and Critical Care Medicine, Shanghai Pudong Hospital, Fudan University, China; Department of Pulmonology, Renji Hospital, Shanghai Jiao Tong University School of Medicine, China; Department of Emergency Medicine, Clinical Center for Bio-Therapy, Department of Biostatistics, and Department of Urology, Shanghai Key Laboratory of Lung Inflammation and Injury, Zhongshan Hospital, Fudan University, China; Department of Emergency Medicine, Clinical Center for Bio-Therapy, Department of Biostatistics, and Department of Urology, Shanghai Key Laboratory of Lung Inflammation and Injury, Zhongshan Hospital, Fudan University, China; Key Laboratory of Systems Biology, Center for Excellence in Molecular Cell Science, Shanghai Institute of Biochemistry and Cell Biology, Chinese Academy of Sciences, China; Department of Emergency Medicine, Clinical Center for Bio-Therapy, Department of Biostatistics, and Department of Urology, Shanghai Key Laboratory of Lung Inflammation and Injury, Zhongshan Hospital, Fudan University, China; Department of Emergency Medicine, Clinical Center for Bio-Therapy, Department of Biostatistics, and Department of Urology, Shanghai Key Laboratory of Lung Inflammation and Injury, Zhongshan Hospital, Fudan University, China; Department of Emergency Medicine, Clinical Center for Bio-Therapy, Department of Biostatistics, and Department of Urology, Shanghai Key Laboratory of Lung Inflammation and Injury, Zhongshan Hospital, Fudan University, China; Department of Emergency Medicine, Clinical Center for Bio-Therapy, Department of Biostatistics, and Department of Urology, Shanghai Key Laboratory of Lung Inflammation and Injury, Zhongshan Hospital, Fudan University, China; Shanghai Public Health Clinical Center, Fudan University, China; Department of Biostatistics, School of Public Health, Key Laboratory of Public Health Safety of Ministry of Education, and Shanghai Institute of Infectious Disease and Biosecurity, School of Public Health, Fudan University, China; Shanghai Institute of Immunology, Department of Immunology and Microbiology, Shanghai Institute of Virology, Key Laboratory of Cell Differentiation and Apoptosis of Chinese Ministry of Education, School of Medicine, Shanghai Jiao Tong University, China; Department of Emergency Medicine, Clinical Center for Bio-Therapy, Department of Biostatistics, and Department of Urology, Shanghai Key Laboratory of Lung Inflammation and Injury, Zhongshan Hospital, Fudan University, China; Department of Emergency Medicine, Clinical Center for Bio-Therapy, Department of Biostatistics, and Department of Urology, Shanghai Key Laboratory of Lung Inflammation and Injury, Zhongshan Hospital, Fudan University, China; Department of Emergency Medicine, Clinical Center for Bio-Therapy, Department of Biostatistics, and Department of Urology, Shanghai Key Laboratory of Lung Inflammation and Injury, Zhongshan Hospital, Fudan University, China; Shanghai Public Health Clinical Center, Fudan University, China; Department of Emergency Medicine, Clinical Center for Bio-Therapy, Department of Biostatistics, and Department of Urology, Shanghai Key Laboratory of Lung Inflammation and Injury, Zhongshan Hospital, Fudan University, China; Department of Emergency Medicine, Clinical Center for Bio-Therapy, Department of Biostatistics, and Department of Urology, Shanghai Key Laboratory of Lung Inflammation and Injury, Zhongshan Hospital, Fudan University, China; Department of Emergency Medicine, Clinical Center for Bio-Therapy, Department of Biostatistics, and Department of Urology, Shanghai Key Laboratory of Lung Inflammation and Injury, Zhongshan Hospital, Fudan University, China; Department of Emergency Medicine, Clinical Center for Bio-Therapy, Department of Biostatistics, and Department of Urology, Shanghai Key Laboratory of Lung Inflammation and Injury, Zhongshan Hospital, Fudan University, China; Department of Emergency Medicine, Clinical Center for Bio-Therapy, Department of Biostatistics, and Department of Urology, Shanghai Key Laboratory of Lung Inflammation and Injury, Zhongshan Hospital, Fudan University, China; Department of Emergency Medicine, Clinical Center for Bio-Therapy, Department of Biostatistics, and Department of Urology, Shanghai Key Laboratory of Lung Inflammation and Injury, Zhongshan Hospital, Fudan University, China; Department of Emergency Medicine, Clinical Center for Bio-Therapy, Department of Biostatistics, and Department of Urology, Shanghai Key Laboratory of Lung Inflammation and Injury, Zhongshan Hospital, Fudan University, China; Department of Emergency Medicine, Clinical Center for Bio-Therapy, Department of Biostatistics, and Department of Urology, Shanghai Key Laboratory of Lung Inflammation and Injury, Zhongshan Hospital, Fudan University, China; Department of Emergency Medicine, Clinical Center for Bio-Therapy, Department of Biostatistics, and Department of Urology, Shanghai Key Laboratory of Lung Inflammation and Injury, Zhongshan Hospital, Fudan University, China; Department of Emergency Medicine, Clinical Center for Bio-Therapy, Department of Biostatistics, and Department of Urology, Shanghai Key Laboratory of Lung Inflammation and Injury, Zhongshan Hospital, Fudan University, China; Department of Biostatistics, School of Public Health, Key Laboratory of Public Health Safety of Ministry of Education, and Shanghai Institute of Infectious Disease and Biosecurity, School of Public Health, Fudan University, China; Department of Emergency Medicine, Clinical Center for Bio-Therapy, Department of Biostatistics, and Department of Urology, Shanghai Key Laboratory of Lung Inflammation and Injury, Zhongshan Hospital, Fudan University, China; Key Laboratory of Systems Biology, Center for Excellence in Molecular Cell Science, Shanghai Institute of Biochemistry and Cell Biology, Chinese Academy of Sciences, China; Department of Emergency Medicine, Clinical Center for Bio-Therapy, Department of Biostatistics, and Department of Urology, Shanghai Key Laboratory of Lung Inflammation and Injury, Zhongshan Hospital, Fudan University, China; State Key Laboratory of Genetic Engineering, Fudan Microbiome Center, School of Life Sciences, Fudan University, China; Bio-Med Big Data Center, Shanghai Institute of Nutrition and Health, Chinese Academy of Sciences, China; Department of Emergency Medicine, Clinical Center for Bio-Therapy, Department of Biostatistics, and Department of Urology, Shanghai Key Laboratory of Lung Inflammation and Injury, Zhongshan Hospital, Fudan University, China; Department of Biostatistics, School of Public Health, Key Laboratory of Public Health Safety of Ministry of Education, and Shanghai Institute of Infectious Disease and Biosecurity, School of Public Health, Fudan University, China; Department of Emergency Medicine, Clinical Center for Bio-Therapy, Department of Biostatistics, and Department of Urology, Shanghai Key Laboratory of Lung Inflammation and Injury, Zhongshan Hospital, Fudan University, China; Department of Biostatistics, School of Public Health, Key Laboratory of Public Health Safety of Ministry of Education, and Shanghai Institute of Infectious Disease and Biosecurity, School of Public Health, Fudan University, China

The emergence of SARS-CoV-2 variant of concern omicron BA.2 led to high infection incidence rates as well as reduced severity of COVID-19 during the Shanghai Spring epidemic of 2022 [1]. We assessed the role of viral pathogenicity and the effectiveness of inactivated vaccines in a retrospective observational study including 10 258 confirmed COVID-19 cases identified between April and June of 2022, from four COVID-19 designated hospitals located in two areas of Shanghai, namely the West Bund and the East Bund, where the populations’ demographic characteristics varied significantly (Table S1, Fig. S1).

The documented weakened pathogenicity of omicron variant BA.2 [2–5] is well demonstrated by a low ratio of inpatients suffering from pneumonia (8.4%, 859/10 258) in this large cohort of confirmed cases (Table S2), including 6.7% and 1.6% for moderate and severe/critical cases (Table S1), respectively. We applied pneumonia as the major criterium to distinguish infection in the upper or lower respiratory tract and to monitor disease severity because it could be unambiguously identified throughout the whole process of the disease, particularly in this mild-case dominated inpatient population, and may reflect either the viral impact that could account for the altering pathogenicity or the vaccination impact related to critical progression of the disease.

To evaluate the viral impact upon pathogenicity, we first investigated the clinical manifestations in inpatients with non-/incomplete vaccination (*n* = 3790, hereafter designated as ‘incomplete vaccination’) as this group was less affected by the protective effect of the vaccine. A majority of the cases were either asymptomatic (18.7%) or mild (diagnosed pneumonia-free, 64.1%) on admission, which mostly featured only upper respiratory tract symptoms (Table S1, Fig. S2) and those percentages were profoundly higher than that caused by the wild type (WT) strain infection [6]. In terms of pneumonia (Table S2) and severe outcomes (Table S3) for inpatients with incomplete vaccination, the incidences were 17.3% and 7.6%, respectively, both much less than that during Wuhan epidemic of 2020 caused by a WT strain in a population absent of any vaccination, that all the inpatients were suffering from pneumonia with 18.6% having severe outcomes [6]. In addition, it should be noticed that, in the incidences of pneumonia, severe outcomes and disease progression increased with age and numbers of comorbidities, regardless of whether the patients had received full/booster vaccinations or not (Fig. 1A–C). Thus, the intrinsic property of omicron BA.2 is likely to account for, to certain extent, the reduced severity of the epidemic. This clinical observation might be underpinned by the mechanistic change of preferred tropism of omicron BA.2 caused by its attenuated infection in the lower respiratory tract but more favorable activity in the upper respiratory tract compared to the previous virus strains due to the highly mutated spike-associated inefficient usage of human transmembrane serine protease 2 for S1/S2 cleavage [2,3,7].

**Figure 1.**
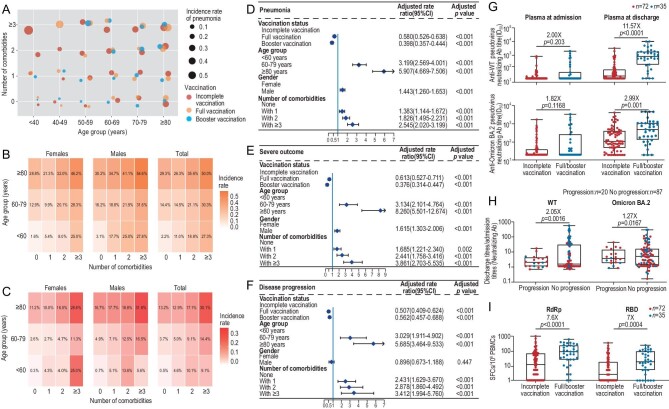
The analyses of virus pathogenicity, vaccination effectiveness, and immune responses among omicron BA.2 infected inpatients of this study. Methodology and primary analysis results are provided in the Supplementary material 1 and 2. The incidence rates of pneumonia in patients with different vaccination coverage distributed among groups with different ages and numbers of comorbidities were demonstrated by a bubble chart (A and Table S2). The incidences of pneumonia and severe outcomes among incompletely-vaccinated patients with different ages and number of comorbidities, demonstrated by heatmap and compared between male and female patients (B, C). Vaccine effectiveness in preventing pneumonia, severe outcomes and disease progression, and the influence of age, gender and number of comorbidities by adjusted rate ratios (D–F and Tables S2, S3, S5). Neutralizing antibody titres to SARS-CoV-2 WT and omicron BA.2 pseudoviruses for both incomplete and full/booster vaccination groups at admission and discharge (G). The ratio of neutralizing antibody titres against WT and omicron BA.2 strains at discharge to that at admission, which is compared in patients with or without disease progression (H). T cell responses (as SFC per million PBMCs) to RBD and RdRp proteins were compared in incomplete and full/booster vaccination groups at admission. SFC represents spot forming cells; PBMCs represent peripheral blood mononuclear cells (I).

We evaluated the protective effect of inactivated vaccines with regard to preventing the disease deterioration of the omicron BA.2 infected inpatients of this study (Supplementary primary analysis results). After adjusting for age, sex and number of comorbidities (Supplementary methods), full and booster vaccinations not only reduced the incidence rate of pneumonia (rate ratio of full *vs* incomplete vaccination 0.580 [95% CI: 0.526–0.638], booster *vs* incomplete vaccination 0.398 [95% CI: 0.357–0.444]) (Fig. 1D, Table S2), and the risk of severe outcomes (Fig. 1E, Table S3); but also prevented disease progression and shortened viral shedding time (Fig. 1F, Fig. S3, Tables S4–6). It is worth emphasizing that vaccination especially benefited the high-risk population (defined by the guidelines of the WHO, refer to the Supplemental methods) by preventing pneumonia, as well as severe outcomes and disease progression (Tables S2–5), whereas, among the non–high-risk subgroup with incomplete vaccination, the incidence rates of pneumonia (Table S2) and severe outcomes (Table S3) were 1.9% and 0.2%, respectively, similar to that of the full/booster vaccinated group, indicating that the effect of vaccination might be negligible in the non–high-risk population. The above results were observed in both the West Bund population and the East Bund population of Shanghai, as well as confirmed by CBPS-IPTW and multivariate Poisson regression models (Tables S7–S12).

To explore the mechanism of vaccine effectiveness, plasma antibodies and T cell responses were determined. For plasma antibody response, the neutralizing antibody (NAb) titre against WT strain at admission in the full/booster vaccination group was 2-fold higher than that in the incomplete vaccination group, then increased to 11.57-fold at discharge; by contrast, it only reached a 2.99-fold difference for NAbs against omicron BA.2 despite the fact it was an omicron BA.2 infection (Fig. 1G). In addition, the booster vaccination group had a significantly lower geometric mean titre (GMT) of NAbs against BA.2 compared to the full vaccination group (Fig. S4). Overall, these data implicate that BA.2 infection in addition to WT inactivated vaccination could evoke a rapid recall response with a phenomenon of ‘Original Antigenic Sin’, as previously reported [8]. Furthermore, we identified that the ratios of NAb GMTs against WT and omicron BA.2 at discharge to that at admission in patients without disease progression were both significantly higher than that in the progression group, which was further supported by plasma IgG GMTs data. However, NAb titres were not significantly associated with the risk of pneumonia (Fig. 1H, Figs S5, S6). Those data demonstrated that inactivated vaccine inoculation establishes pre-existing immune responses which could be rapidly recalled for memory B cells. In this study, increased anti-receptor binding domain (RBD) IgG and NAb titres in patients who received full/booster vaccinations are in accordance with the clinical findings that inactivated vaccine inoculations prevented the development of pneumonia and disease progression.

For T cell responses, we employed the viral RBD fragment as the stimulating antigen due to its universal presence in all of the available COVID-19 vaccines as the protective immunogen and enrichment with genetic mutations along the pandemic [9], which provided an identical platform to gauge T cell responses elicited by different COVID-19 vaccines and to determine the immune escape. We also included the viral RNA-dependent RNA polymerase (RdRp) in our assay, because it not only is one of the most conserved non-structured proteins of the coronavirus [10], but also can be identified in the inactivated vaccines (Table S13, Fig. S7, Supplementary methods). Although the RdRp-specific T cell response was shown to be cross-protective in a mouse vaccine-and-challenge model [11], its clinical impact is yet to be determined.

Notably, T cell responses targeting RBD and RdRp antigens at admission were profoundly higher in patients with full/booster vaccinations than that in the incomplete vaccination group (Fig. 1I). In addition, patients without pneumonia or disease progression had higher anti-RBD and RdRp T cell responses at discharge (Figs S5, S6). Thus, this study provided the first line of evidence that sustained T cell responses may facilitate the rapid increase of antibody responses after omicron BA.2 breakthrough infection [12]. It may also underline the fact that with the emergence of the omicron variant of concern, the inactivated vaccination was as effective as that of the mRNA vaccination in preventing severe disease and death [13]. In other words, T cell responses, particularly for patients with full/booster inactivated vaccinations and infected by highly mutated viruses such as omicron BA.2, may play a more important role than NAbs in reducing the risk of pneumonia and disease progression.

Omicron BA.2 is a critical variant of concern along the COVID-19 pandemic. Systematic molecular immunology, virology and animal model studies [2–5] revealed that large numbers of variations in the spike protein of omicron BA.2 dramatically changed its antigenicity and cell entry mechanisms, underlying its rapid global spread and altered pathogenicity. Based on the large inpatient cohort of the 2022 Shanghai Spring epidemic, our findings validate that the low pathogenicity of the omicron BA.2 variant is the most important contributor accounting for the reduced clinical severity, clearly observed in the non–high-risk population and is probably related to the change of its infection tropism. Our data support that the incidence of pneumonia is the most reliable criterium for monitoring the disease severity and progression. In addition, a vaccination strategy with inactivated vaccines provides protection from occurrence of pneumonia, severe outcomes and disease progression, and particularly benefits the high-risk population. Meanwhile, T cell responses, both elicited by RBD and RdRp, confer the important protective effects of the inactivated virus vaccines in addition to NAbs.

Abbreviations CBPS-IPTW: covariate-balancing propensity score inverse probability treatment weighting
COVID-19: coronavirus disease 2019
GMTs: geometric mean titres
NAb: neutralizing antibody
PBMCs: peripheral blood mononuclear cells
RBD: receptor binding domain
RdRp: RNA-dependent RNA polymerase
RT-PCR: real-time polymerase reaction chain
SARS-CoV-2: severe acute respiratory syndrome coronavirus 2
SFC: spot forming cells
WHO: World Health Organization
WT: wild type

## Supplementary Material

nwae011_Supplemental_File
