# Supplementary material for: Reduced clinical severity during 2022 Shanghai Spring epidemic of SARS-CoV-2 omicron BA.2 variant infection—an integrated account of virus pathogenicity and vaccination effectiveness
Source: Natl Sci Rev. 2024 Jan 15;11(4):nwae011. doi: 10.1093/nsr/nwae011 (PMC11065342; doi:10.1093/nsr/nwae011)
Supplement: nwae011_Supplemental_File [file nwae011_supplemental_file.docx]

Reduced clinical severity during 2022 Shanghai Spring epidemic of SARS-CoV-2 omicron BA.2 variant infection - An integrated account of virus pathogenicity and vaccination effectiveness

**Supplementary materials**

**1. Primary analysis results**..............................................................................................................1

**2. Methods**.......................................................................................................................................3

**3. Figures and tables**

**Figure S1** Flow chart of study population screening......................................................................11

**Figure S2** Symptoms among inpatients with different vaccination status......................................12

**Figure S3** Forest plots of the vaccine effectiveness in reducing viral shedding time.....................13

**Figure S4** Plasma antibody responses for SARS-CoV-2 WT and omicron BA.2 strain at admission and discharge..........………................……….......…......................................................................14

**Figure S5** Plasma antibody and T cell responses associated with the risk of pneumonia..............15

**Figure S6** Plasma antibody and T cell responses associated with the risk of disease progression….16

**Figure S7** The Spectrum of RdRp in inactivated vaccines via proteomics and LC-MS/MS analysis...17

**Figure S8** Vaccination status in COVID-19 inpatients among different age groups……..............18

**Figure S9** Changes in disease severity of infection from hospital admission to discharge............19

**Table S1** Baseline and clinical characteristics of study population    ............................................20

**Table S2**Inactivated vaccines prevented pneumonia in COVID-19 inpatients..............................23

**Table S3**Inactivated vaccines prevented severe outcomes in COVID-19 inpatients.....................24

**Table S4**Baseline and Clinical characteristics of COVID-19 inpatients who had disease progression ......................................................................................................................................25

**Table S5** Inactivated vaccines prevented disease progression in COVID-19 inpatients ...............26

**Table S6**Inactivated vaccines reduced viral shedding time of SARS-CoV-2................................27

**Table S7** Baseline and clinical characteristics of West Bund population.......................................28

**Table S8** Baseline and clinical characteristics of East Bund population........................................30

**Table S9** Effectiveness of inactivated vaccines in preventing pneumonia in COVID-19 inpatients validated in West Bund population and East Bund population.......................................................32

**Table S10** Effectiveness of inactivated vaccines in preventing severe outcomes in COVID-19 inpatients validated in West Bund population and East Bund population.......................................33

**Table S11** Effectiveness of inactivated vaccines in preventing disease progression in COVID-19 inpatients validated in West Bund population and East Bund population.......................................34

**Table S12** Effectiveness of inactivated vaccines in reducing viral shedding time in COVID-19 inpatients validated in West Bund population and East Bund population.......................................35

**Table S13** Peptides identification of RdRp in inactivated vaccines...............................................36

**Table S14** STROBE Statement.......................................................................................................37

**Table S15** Definitions of comorbidities..........................................................................................40

**Table S16** Clinical severity classification.......................................................................................41

**Table S17** Definitions of outcomes.................................................................................................42

**Primary analysis results**

Baseline and clinical characteristics of all inpatients are listed in **Table S1.** Among the participants, 36.9% (3790/10258) inpatients had incomplete vaccination, 27.5% (2819/10258) had full vaccination and 35.6% (3649/10258) had booster vaccination. High-risk populations (47.8%, 4905/10258) had a low vaccination coverage, only 20.2% and 24.4% of whom received full or booster vaccination respectively. The median age was 69 years old (IQR: 43-82) for incomplete vaccination group, and more than half (52.9%, 2005/3790) had comorbidities. Also, the numbers of inpatients with full/booster vaccination decreased among the elder population (**Figure S8)**. On admission, most of cases were asymptomatic (22.7%) or mild (68.9%), followed by some moderate cases (6.7%)，and only 1.6% were severe or critical cases. And more inpatients in incomplete vaccination group (655/3790, 17.3%) were diagnosed as moderate or severe/critical cases than those in full vaccination group or booster vaccination group (114/2819, 4.0%; 90/3649, 2.5%).

We particularly explored the clinical manifestations in inpatients with incomplete vaccination, which reflected the pathogenicity of omicron BA.2 without the influence of vaccines. Symptoms in upper respiratory tract were the common clinical symptoms in COVID-19 infections, including cough (36.0%), expectoration (19.9%) and sore throat (11.8%). Contrarily, symptoms in lower respiratory tract were comparatively rare, such as chest tightness (2.1%), shortness of breath (2.0%) and chest pain (0.4%) (**Table S1, Figure S2**). Most cases were asymptomatic (18.7%, 707/3790) or mild (64.1%, 2428/3790) on admission. The proportion of moderate cases and severe/critical cases were 13.7% (518/3790) and 3.6% (137/3790), and the rate of pneumonia on admission and severe outcomes during hospitalization were even lowered to only 2.2% and 0.5% respectively, among inpatients aged < 60 years old and without comorbidities. The incidence of pneumonia and severe outcomes increased with age and numbers of comorbidities (**Figure 1B, 1C, Table S2, Table S3**).

Pneumonia is a critical outcome of omicron BA.2 infection that actually added to healthcare burden. It was demonstrated that the risk of developing pneumonia increased along with age and numbers of comorbidities (**Figure 1A)**. Male inpatients also had a higher risk of pneumonia. Full (RR_CBPS-IPTW_ = 0.580 [95% CI: 0.526-0.638]) and booster vaccination (RR_CBPS-IPTW_ = 0.398 [95% CI: 0.357-0.444]) reduced risk of pneumonia, which were consistent with multivariate poisson regression model and CBPS-IPTW analysis. Notably, high-risk subgroup especially benefited from this protective effect (full vaccination: RR_CBPS-IPTW_ = 0.585 [95% CI: 0.528-0.648], booster vaccination: RR_CBPS-IPTW_ = 0.399 [95% CI: 0.355-0.447]) (**Figure 1D, Table S2**).

Then, we investigated into whether inactivated vaccines prevented inpatients from disease progression **(Figure S9)**. A total of 199 (5.5%) patients progressed to the severer type of infection during hospitalization. And inpatients with disease progression mostly did not receive full vaccination (72.9%) (**Table S4**). Inactivated vaccines could prevent disease progression (full vaccination: RR_CBPS-IPTW_ = 0.507 [95% CI: 0.409-0.624], booster vaccination: RR_CBPS-IPTW_ = 0.562 [95% CI: 0.457-0.688]) **(Figure 1F, Table S5)**. Moreover, we focused on whether vaccination could protect inpatients from severe outcomes. In total, 344 out of 10258 (3.4%) inpatients progressed to severe outcomes, 287 of whom (83.4%) were in the incomplete vaccination group and 334 (97.1%) were from high-risk population. Our results showed that the full/booster vaccination reduced the development of severe outcomes (full vaccination: RR_CBPS-IPTW_ = 0.613 [95% CI: 0.527-0.711], booster vaccination: RR_CBPS-IPTW_ = 0.376 [95% CI: 0.314-0.447]). The high-risk subgroup showed the similar results in preventing disease progression and severe outcomes. Both CBPS-IPTW method and multivariate poisson regression model came to the same conclusions (**Figure 1E, Table S3**).

Viral shedding time reflects the duration of infectiousness of SARS-CoV-2. The median days of viral shedding time were 13 (IQR: 9-17) in the incomplete vaccination group, 10 (IQR: 7-14) in the full vaccination group, and 10 (IQR: 7-13) in the booster vaccination group. Vaccination effectively reduced the viral shedding time (full vaccination: HR_CBPS-IPTW_ = 1.203 [95% CI: 1.126-1.285], booster vaccination: HR_CBPS-IPTW_ = 1.207 [95% CI: 1.120-1.300]). Subgroup analyses validated that vaccination could reduce the viral shedding time for both high-risk and non-high risk population (**Figure S3, Table S6**).

We further validated the effects of inactivated vaccines in preventing pneumonia, disease progression, severe outcomes and reducing viral shedding time in West Bund and East Bund population respectively. In addition to the whole population, the protective effects of inactivated vaccines were confirmed in different areas of Shanghai, both West Bund and East Bund population **(Table S7-12)**.

Plasma antibodies against both WT and omicron BA.2 strains at admission and at discharge of 107 inpatients were determined, among whom 67.3% (72/107) inpatients received incomplete vaccination and 32.7% (35/107) were from full or booster group. For WT strain specific antibodies, plasma RBD-binding IgG in the full/booster vaccination group was 2-fold higher than that in incomplete vaccination group (p = 0.0537) at admission, then increased to a 7.14-fold (IgG: 13805 [95% CI: 7705-24735] *vs* 1777 [95% CI: 1183-2669], p < 0.0001) at discharge. Moreover, nAb titres in the full/booster vaccination group showed an increase from 2-fold at admission (p = 0.203) to 11.57-fold higher at discharge (613.5 [95% CI: 335.5-1122] *vs* 53.5 [95% CI: 39.12-73.15], p < 0.0001) in comparison with that in incomplete group. Interestingly, plasma IgG and nAb against omicron BA.2 displayed a similar trend but a much less increase (**Figure 1G, Figure S4**). Then, we explored the effectiveness of antibodies in preventing pneumonia. Only plasma IgG GMTs were approximately 2-fold higher in inpatients without pneumonia than that in inpatients with pneumonia for two strains (**Figure S5**). In order to determine whether the increasing ratio of antibodies for individuals at discharge and at admission are related to disease progression, we compared the increasing ratio in 20 out of 107 inpatients who progressed to the severer infection types during hospitalization with the left 87 inpatients who did not. As shown in **Figure 1H and Figure S6,** significant differences were observed in plasma NAb GMTs for both WT and BA.2, which was not shown for IgG GMTs. Additionally, we also measured the T cell responses recalled from the preexisting immune reaction. Anti-RBD and RdRp T cell responses were significantly higher in inpatients who received full/booster vaccination than in incomplete vaccination group, which means T cell responses maintained after vaccination and this sustained T cell responses might be dedicated to the rapid restoration of antibody responses after infection. In addition, robust anti-RBD and RdRp T cell responses at discharge were significantly associated with the lower risk of pneumonia and disease non-progression. (**Figure 1I and Figure S5, S6**).

**Methods**

**Study design and population**

We assessed the virus pathogenicity and effectiveness of inactivated SARS-CoV-2 vaccines in this retrospective observational study. The study population was from four COVID-19 designated hospitals located in two areas of Shanghai: (1) West Bund population: Zhongshan hospital and Minhang Gymnasium COVID-19 designated hospital, Fudan University located to the west of Huangpu River; (2) East Bund population: Renji hospital, Shanghai Jiaotong University, School of Medicine, and Pudong hospital, Fudan University located to the east of Huangpu River. The study population included Shanghai residents with laboratory-confirmed SARS-CoV-2 infection by real-time polymerase reaction chain (RT-PCR) tests between April 2022 and June 2022. Inpatients were excluded if they had following conditions: (1) under 3 years old, (2) with unclear SARS-CoV-2 vaccination history, (3) having received other types of vaccines rather than inactivated vaccines, (4) the interval between the confirmation of infection and admission was over 14 days, (5) HIV infection (viral load over 400 copies/mL) or suspected/confirmed active systemic infection, (6) pregnancy or breastfeeding, (7) comorbidities requiring hospitalization/surgery, (8) considered life threatening under 30 days, (9) receipt of convalescent COVID-19 plasma (**Figure S1**). Then we classified enrolled inpatients into high-risk subgroup and non-high-risk subgroup. According to the guidelines of World Health Organization (WHO) ^1^, the high- risk subgroup is defined as people who are older than 60 years or have health conditions including lung or heart disease, diabetes, or conditions that affect their immune system.

Furthermore, we selected inpatients admitted in Zhongshan hospital between May 1^st^ 2022 and June 15^th^ 2022 for antibody response assessment. The inclusion criteria for these participants are as follows: (1) willing to donate peripheral blood sample; (2) in-hospital length of stay ≥ 7 days; (3) the interval between the day of admission and first donation was ≤ 7 days; (4) the interval between the day of first donation and last donation ≥ 7 days; (5) without exclusion criteria mentioned above.

The study was approved by the Ethical Committee of Zhongshan hospital, Fudan University (B2022-244R), Shanghai Pudong hospital, Fudan University (WZ-22) and Renji hospital, Shanghai Jiaotong University, School of Medicine (RA-2022-601). The study was registered on www.chictr.org.cn (ChiCTR2200060003). And the Strengthening the Reporting of Observational Studies in Epidemiology (STROBE) checklists were used to guide transparent reporting (**Table S14**).

**Procedures**

Extensive PCR testing for SARS-CoV-2 was done for every Shanghai resident between April 2022 and June 2022. Symptomatic or high risk COVID-19 cases were admitted to COVID-19 designated hospitals. Inpatients received daily pharyngeal swab SARS-CoV-2 PCR test during hospitalization, and virus clearance was considered when two consecutive negative tests with an interval of over 24 hours were reported (cycle threshold value large than 35 in at least ORF1ab and N genes).

Baseline characteristics and clinical information were acquired from the hospital electronic medical records. Definitions for the comorbidities are listed in **Table S15**. And vaccination history was obtained from the Shanghai Group Immunization System, which included comprehensive vaccination information of all vaccines recipients, such as the type, manufacturer, date, and doses of vaccines. In this study, the manufacturers of inactivated vaccines included CoronaVac, Sinopharm/BBIBP-CorV, and Sinopharm/WIBP-CorV. Meanwhile, peripheral blood samples were collected from corresponding participants for antibody response assessment and the titre changes of binding and neutralizing antibody were measured.

**Infections and Outcomes**

The diagnosis and clinical severity classification were according to the ninth version of the *Chinese Clinical Guidance for COVID-19 Pneumonia Diagnosis and Treatment*^2^. Generally, cases were classified into asymptomatic, mild, moderate, severe and critical infections. Inpatients who had signs of pneumonia would be diagnosed as moderate, severe or critical infections (**Table S16**).

Vaccination status was defined as: (1) incomplete vaccination (0/1 dose): no history of inactivated SARS-CoV-2 vaccine inoculation; one dose of vaccine inoculation, or the second dose received within 14 days before the last SARS-CoV-2 exposure; (2) full vaccination (2 doses): two doses of vaccines inoculation and the most recent dose received > 14 days before the last SARS-CoV-2 exposure, or the booster dose received within 7 days before the last SARS-CoV-2 exposure; (3) booster vaccination (3 doses): booster shot received > 7 days before the last SARS-CoV-2 exposure.

We compared the effectiveness of inactivated vaccines in four study endpoints: (1) pneumonia; (2) disease progression; (3) severe outcomes and (4) viral shedding time (**Table S17**). In addition to full population analyses, all study results were validated in subgroup analyses which included West Bund population or East Bund population.

**Antibody response measurement**

Binding antibodies were measured by enzyme linked immunosorbent assay (ELISA) to quantify plasma IgG binding to RBD of wild type (WT) and Omicron BA.2. ELISA endpoint titres were defined as the highest dilution that yields an absorbance of 2-fold greater than background value. In brief, 96-well ELISA plates were coated with 0.5 mg/mL of recombinant SARS-CoV-2 RBD protein at 4 °C overnight. After washing the plates with PBS containing 0.5% Tween-20 (PBST) and blocking with 5% non-fat milk in PBST (PBST/5% milk) for 2 h at room temperature (RT), a 2-fold dilutions series (generally staring from 1:100) of human plasma samples were added, followed by 3 h incubation at RT. The plates were subsequently extensively washed with PBST before subjecting to total IgG measurement. For total IgG measurement, a dilution of 1:5000 of HRP-conjugated goat anti-human IgG was applied in PBST/5% milk. After 1 h incubation at RT, the plates were extensive washed with PBST before addition of the substrate OPD (one SIGMAFAST OPD tablet (Sigma, SLCC0308) in 20 mL of deionized water). The reactions lasted for 5 min at RT and were terminated by adding 1M H2SO4, followed by reading at OD490 with a Synergy Microplate Reader (Bio-Tek, Winooski, VT).

Neutralizing antibody responses were quantified by pseudovirus (WT and omicron BA.2) based neutralization assay. The neutralizing ID50 (50% inhibitory doses) titres were calculated from the highest dilution resulting in a 50% reduction in relative light units (RLUs) relative to virus control wells after background (RLUs of no virus wells) subtraction. Briefly, HEK 293T cells were co-transfected with pNL4-3.Luc.R-E- (NIH AIDS Reagent Program, cat#3418) and pcDNA3 plasmid encoding SARS-CoV-2 WT or Omicron BA.2 spike using TurboFect reagent (Thermo Scientific, cat#R0531). Twelve hours post transfection, culture medium was refreshed with complete D10 medium for additional 48 h incubation. The pseudovirus-containing supernatants were then harvested, cleared by centrifugation, and stored at -80 °C as single-use aliquots after filtering through 0.45μm. For each pseudovirus stock, a titration assay was performed using an aliquot to determine the dilution needed for the neutralization assay. To assess plasma neutralizing activity against SARS-CoV-2, serial 50-μL dilutions of heat-inactivated plasma were made with D10 medium and mixed with equal volume of diluted pseudovirus. After incubation at 37 °C for 1 h, the plasma-pseudovirus mixtures were transferred to a 96-well plate containing hACE2-293T cells, which were pre-seeded at 2×104 cells per well and allowed to grow for 12 h. The plates were incubated for 48 h at 37°C in presence of 5% CO2 and luciferase activities were then measured using Bright-Glo™ Luciferase Assay System (Promega) on a luminometer (Promega GloMax 96).

**T cell response measurement**

T cell responses were analyzed using a human IFN-γ ELISpot assay set (BD Bioscience, #551849) following the manufacture’s protocol. In brief, 96-well ELISpot plates were pre-coated with an anti-human IFN-γ antibody (5 μg/mL, 100 μL) overnight at 4 °C. After rinsing with RPMI-1640 medium (Corning, 10–040-CVR) containing 10% fetal bovine serum (FBS) (BI, 04–001-1acs) and 1% penicillin-streptomycin (PS) (Corning, 30–002-CI) (R10), plates were blocked with R10 for 2 h at room temperature (RT) before adding to each well 2×105 of PBMCs, followed by stimulation with RBD and RdRp proteins. Each assay was performed in duplicate. Protein stimulation lasted 24 h as plates were placed in a humidified 5% CO2 incubator at 37 °C. The plates were washed before biotinylated anti-human IFN-γ antibody (2 μg/mL, 100 μL) was added for 2 h RT incubation. Next, streptavidin conjugated horseradish peroxidase was added at 1:100 dilution and incubated at RT for 1 h. The plates were washed again and subjected to spot development with AEC substrate reagent (BD Bioscience, #551951), with the reaction being stopped by water rinsing and the plates subsequently allowed to dry 24 h in darkness. Plate images were captured with a Biospot plate reader (ChampSpot III, Beijing SageCreation Science Co.,Ltd) and analyzed for spot-forming cells (SFCs) counts.

**Sample preparation for proteomics analysis**

Sample in solution were performed by chloroform-methanol precipitation for proteomic sample preparation. In detail, 10 μL protein solution were mixed with 90 μL lysis buffer (8M Urea, 10 mM TCEP (tris(2-carboxyethyl) phosphine), 40 mM CAA (chloroacetamide),100 mM Tris-HCl, pH 8.0) for 30 min at 37 °C for protein reduction and alkylation. Then, 400 μL methanol and 100 μL chloroform were added, vortexed and following 300 μL H_2_O, finally shaking for 30 s at room temperature. After centrifugation at 15 000 g for 10 min at 4 °C, the supernatant was discarded. Then, 500 μL chilled methanol were added, vortexed and centrifuged at 15 000 g for 10 min at 4 °C. The methanol wash step was repeated twice. Thereafter, precipitated proteins were digested with 2 μg trypsin (Promega) in 100 μL 50 mM ammonium bicarbonate overnight at 37 °C, and peptides were collected by centrifugation at 15 000 g for 10 min at 4 °C. After peptide concentration measurement by BCA Protein Assay (Pierce, Thermo Scientific), a total of 5 μg peptides were desalted before LC-MS/MS analysis.

**LC-MS/MS analysis**

The online reverse-phase (RP) chromatography was performed on a nanoElute System coupled with timsTOF Pro mass spectrometer (Bruker Daltonik GmbH). Peptides (200 ng) were separated on a 20 cm column (i.d. 75 μm) packed in-house with the RP materials ReproSil-Pur C18-AQ, 1.9 μm resin (Dr. Maisch GmbH, Germany). Column was heated at 55 °C and connected with a CaptiveSpray ion source (Bruker Daltonik GmbH). The RP separation gradient flow rate was 400 nl/min for 180 min, including 156 min and 9 min with a linear gradient from 4% to 28% and from 28% to 35% of acetonitrile with 0.1% (v/v) formic acid, respectively.

The parallel accumulation-serial fragmentation (PASEF) method was defined with a full scan (m/z 100 – 1 700, resolution of 60 000), and the singly-charge precursors were excluded by the m/z-ion mobility position. The isolation window of quadrupole was set to 2 Th for m/z < 700 and 3 Th for m/z ≥ 700, and the collision energies varied between 20 and 59 eV depending on precursor mass and charge. A total of 10 PASEF scans (using an accumulation and ramp time of 100 ms) per PASEF scan included on average 12 MS/MS scans, with the resolution of 50 000 (m/z 100 – 1 700).

**Database searching**

All .d raw MS data were analyzed with the MaxQuant software (v2.2.0.0) by default settings. Briefly, a minimum peptide length of 7 amino acids were required, and a false discovery rate (FDR) of 0.01 for both proteins and peptides were applied. MS/MS spectra were searched by the Andromeda search engine incorporated in the MaxQuant software against the uniprot human database (202107, 20612 items) and the database of severe acute respiratory syndrome coronavirus (SARS-CoV), where 69 reviewed protein items included (202206). Trypsin/P was chosen as enzyme specificity, and cysteine carbamidomethylation were selected as a fixed modification, while protein N-terminal acetylation and methionine oxidation were selected as variable modifications. Maximally two missed cleavages were allowed. Initial mass deviation for the precursor and fragment ions was 10 ppm.

**Statistical analysis**

For the pneumonia, disease progression and severe outcomes during hospitalization, poisson regression model with person-time as an offset was applied to compare the number of events among vaccine groups and derive rate ratios (RRs) with 95% CIs. Multivariate poisson models were also applied to adjust for the unbalanced baseline covariates, including age groups, sex, and number of comorbidities. To help account for the nonrandomized stands application, covariate-balancing propensity score inverse probability treatment weighting (CBPS IPTW) method were used to reduce the effects of confounding. The propensity scores were estimated using a logistic regression model with included all characteristic covariates collected under covariate balancing constraint. The predicted probabilities from the PS model were obtained to calculate the inverse-probability weight (IPTW) that ensured covariate balance.

Cox regression models were used to estimate the association between vaccine groups and time to event endpoint of the viral shedding time. Multivariate cox regression models were also applicated to adjust covariates. CBPS IPTW method was applied to reduce the confounders. The results which are consistent in multivariate analysis and CBPS IPTW were considered to be robust.

For the antibodies measurement analysis, pseudovirus NAb and anti-RBD IgG geometric mean titres (GMTs) were evaluated at admission and discharge. We calculated 95% CIs for GMTs by back transforming the 95% CI for log antibody titres. Serum with undetectable antibody titres (below the lower limit of detection, LoD) were given the value of half the LoD for GMT calculations. T cell responses were demonstrated by (SFCs) counts and the data were represented as median with interquartile range (IQR). Comparison of antibody titres and T cell responses between different groups was done using *t test* and two-tailed *Mann*-*Whitney U-test*. The association of neutralizing Ab titres, the ratio of neutralizing Abs titres (at discharge/at admission), T cell responses and the risk of pneumonia and disease progression was done using *Pearson* correlation analysis.

Statistical analyses were performed using R version 4.2.1 (the R foundation) and Graphpad Prism 8.0. p values were two-sided, and p < 0.05 was considered statistically significant.

**References**

1. Organization WH. COVID-19: vulnerable and high risk groups. https://www.who.int/westernpacific/emergencies/covid-19/information/high-risk-groups (12/17 2022, date last accessed).

2. China NHCotPsRo. Chinese Clinical Guidance for COVID-19 Pneumonia Diagnosis and Treatment (9th version). In: China, NHCotPsRo (ed.)2022.


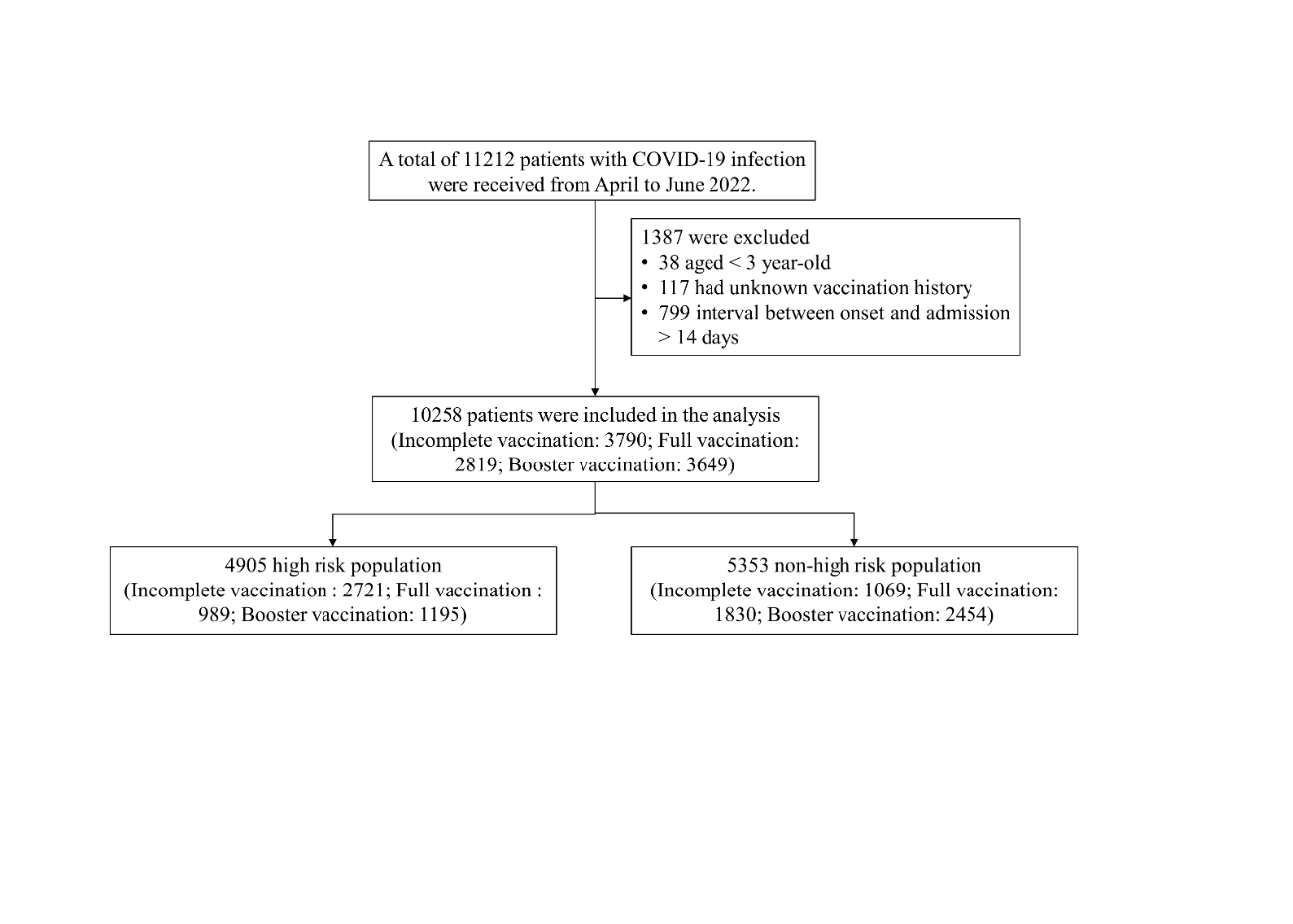
**Figure S1 Flow chart of study population screening.**


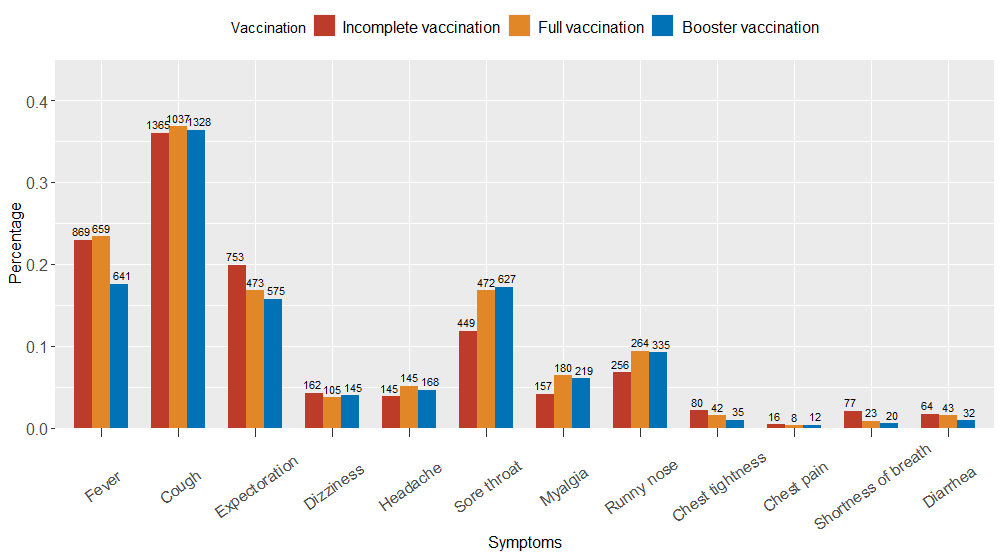


**Figure S2 Symptoms among inpatients with different vaccination status.**

**
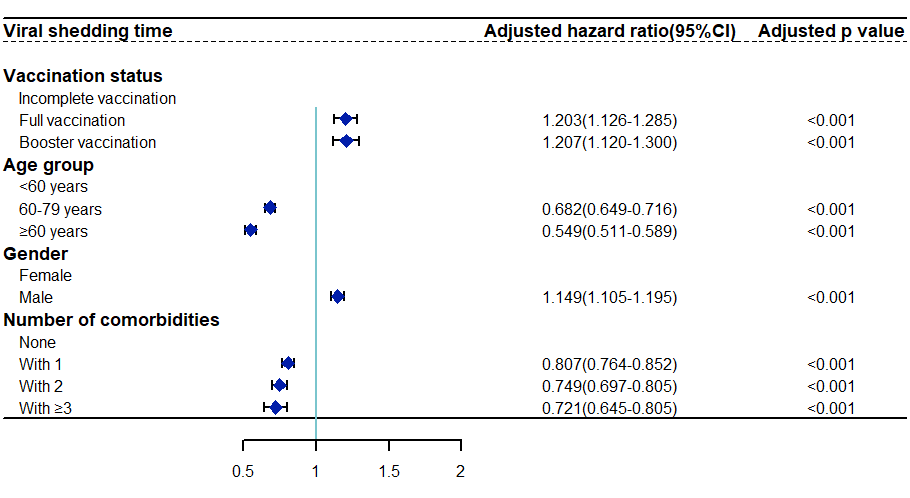
**

**Figure S3** **Forest plots of the vaccine effectiveness in reducing viral shedding time.**

**Figure S4 Plasma antibody responses for SARS-CoV-2 WT and omicron BA.2 strain at admission and discharge.**

**(A)** Geometric mean (95% CI) anti-RBD IgG ELISA unit responses to WT and omicron BA.2 recombinant SARS-CoV-2 protein antigens at admission and discharge. (**B**) The ratio of anti-RBD IgG titres and neutralizing antibody titres against WT and omicron BA.2 strains at discharge to admission. **(C)** Neutralizing antibodies showing response at an inhibitory concentration of more than 50% to SARS-CoV-2 pseudoviruses (WT and omicron BA.2) at discharge.

**Figure S5 Plasma antibody and T cell responses associated with the risk of pneumonia.**

Geometric mean titres (95% CI) anti-RBD IgG ELISA unit responses to WT and omicron BA.2 recombinant SARS-CoV-2 protein antigens at admission and discharge (**A**) and the ratio of discharge titres to admission titres (**B**). Neutralizing antibodies showing response at an inhibitory concentration of more than 50% to SARS-CoV-2 pseudoviruses (WT and omicron BA.2) at admission and discharge (**C**) and the ratio of discharge titres to admission titres (**D**). **(E)** T cell responses (as SFC per million PBMCs) to RBD and RdRp proteins were compared for both pneumonia and no pneumonia groups at discharge. Data were represented as median with interquartile range (IQR). P value was determined by Mann-Whitney U test.

**Figure S6 Plasma antibody and T cell responses associated with the risk of disease progression.**

(**A**) Geometric mean titres (95% CI) anti-RBD IgG ELISA unit responses to WT and omicron BA.2 recombinant SARS-CoV-2 protein antigens at admission and discharge. (**B**) Neutralizing antibodies showing response at an inhibitory concentration of more than 50% to SARS-CoV-2 pseudoviruses (WT and omicron BA.2) at admission and discharge. **(C)** T cell responses (as SFC per million PBMCs) to RBD and RdRp proteins were compared for both progression and no progression groups at discharge. Data were represented as median with interquartile range (IQR). P value was determined by Mann-Whitney U test.


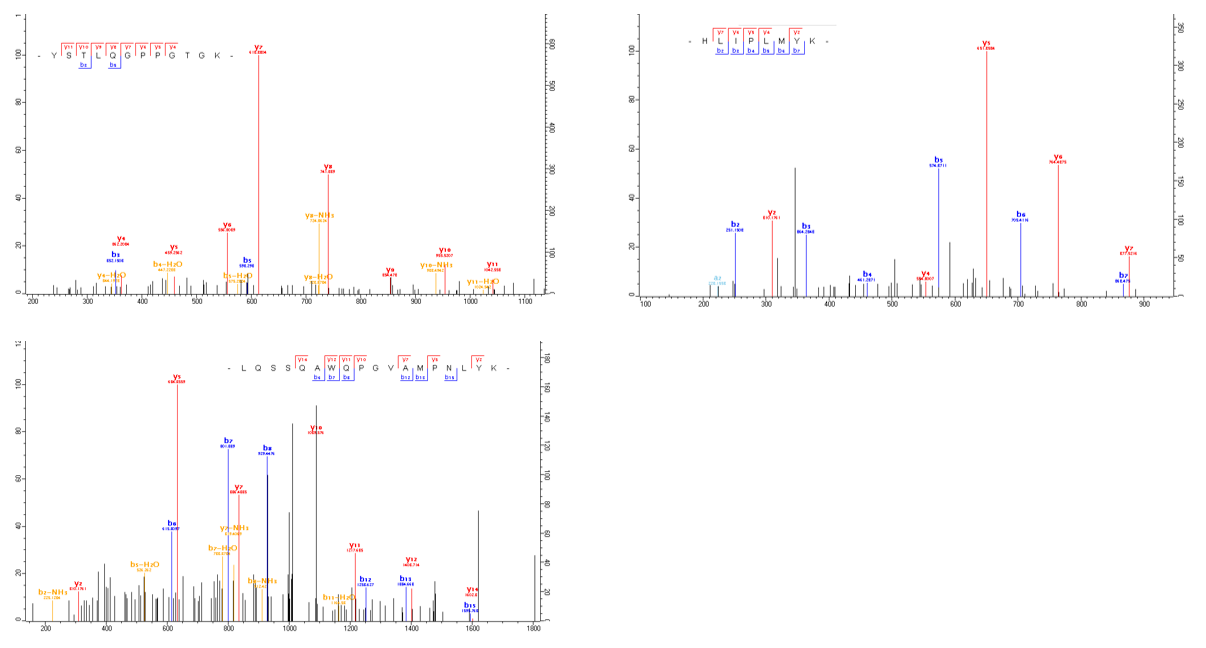


**Figure S7 The Spectrum of RdRp in inactivated vaccines via proteomics and LC-MS/MS analysis.**

**
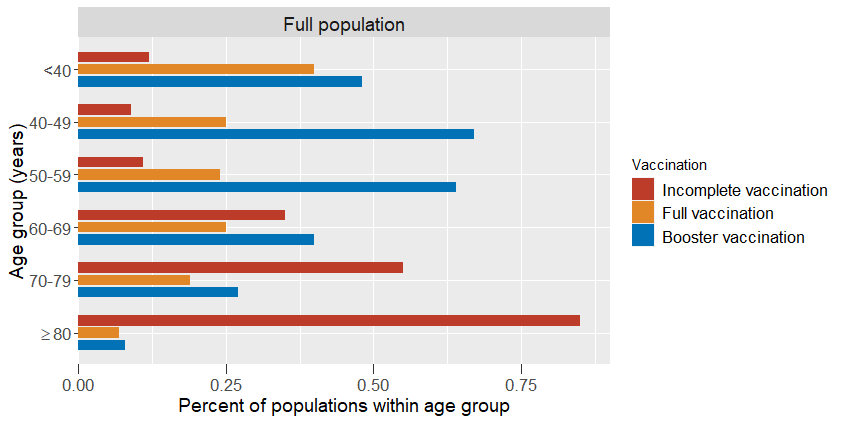
**

**Figure S8 Vaccination status in COVID-19 inpatients among different age groups.**

**
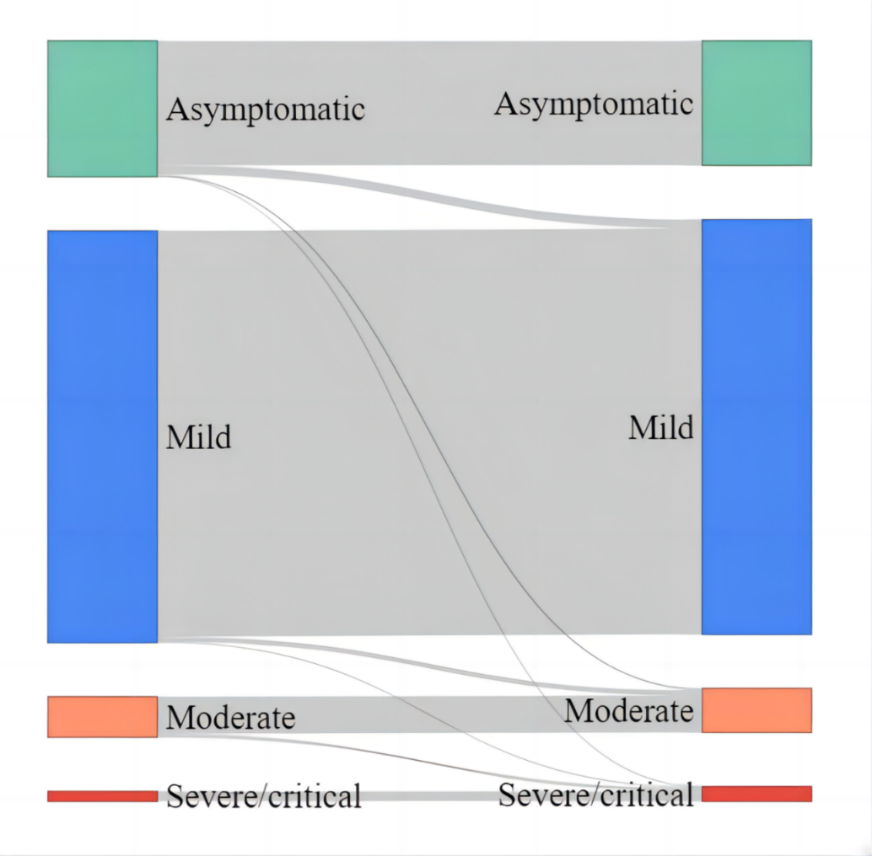
**

**Figure S9 Changes in disease severity of infection from hospital admission to discharge.**

**Table S1 Baseline and clinical characteristics of study population**

|  | **Full population**  **(N=10258)** | | | | | | |  | **High-risk population**  **(N=4905)** | | | | | | |
| --- | --- | --- | --- | --- | --- | --- | --- | --- | --- | --- | --- | --- | --- | --- | --- |
|  | **Incomplete vaccination**  **(N=3790)** |  | **Full vaccination**  **(N=2819)** | | **Booster vaccination**  **(N=3649)** | | **Vaccination^#^**  **(%)** |  | **Incomplete vaccination**  **(N=2721)** | | **Full vaccination**  **(N=989)** |  | **Booster vaccination**  **(N=1195)** |  | **Vaccination^#^**  **(%)** |
| **Characteristics** |  |  |  |  |  |  |  |  |  |  |  |  |  |  |  |
| Median age (IQR) - yr | 69 (43, 82) |  | 44 (31, 62) |  | 50 (37, 59) |  | - |  | 76 (67, 86) |  | 67 (61, 73) |  | 65 (60, 71) |  | - |
| Age group - no. (%) |  |  |  |  |  |  |  |  |  |  |  |  |  |  |  |
| <40 yr | 885 (23.4) |  | 1222 (43.3) |  | 1084 (29.7) |  | 72.3 |  | 48 (1.8) |  | 37 (3.7) |  | 38 (3.2) |  | 61.0 |
| 40 to 49 yr | 154 (4.1) |  | 362 (12.8) |  | 706 (19.3) |  | 87.4 |  | 46 (1.7) |  | 46 (4.7) |  | 70 (5.9) |  | 71.6 |
| 50 to 59 yr | 247 (6.5) |  | 457 (16.2) |  | 956 (26.2) |  | 85.1 |  | 123 (4.5) |  | 128 (12.9) |  | 184 (15.4) |  | 71.7 |
| 60 to 69 yr | 642 (16.9) |  | 417 (14.8) |  | 533 (14.6) |  | 59.7 |  | 642 (23.6) |  | 417 (42.2) |  | 533 (44.6) |  | 59.7 |
| 70 to 79 yr | 729 (19.2) |  | 266 (9.4) |  | 283 (7.8) |  | 43.0 |  | 729 (26.8) |  | 266 (26.9) |  | 283 (23.7) |  | 43.0 |
| ≥80 yr | 1133 (29.9) |  | 95 (3.4) |  | 87 (2.4) |  | 13.8 |  | 1133 (41.6) |  | 95 (9.6) |  | 87 (7.3) |  | 13.8 |
| Gender - no. (%) |  |  |  |  |  |  |  |  |  |  |  |  |  |  |  |
| Female | 2070 (54.6) |  | 1503 (53.3) |  | 1759 (48.2) |  | 61.2 |  | 1447 (53.2) |  | 528 (53.4) |  | 555 (46.4) |  | 42.8 |
| Male | 1720 (45.4) |  | 1316 (46.7) |  | 1890 (51.8) |  | 65.1 |  | 1274 (46.8) |  | 461 (46.6) |  | 640 (53.6) |  | 46.4 |
| Number of comorbidities - no. (%) | |  |  |  |  |  |  |  |  |  |  |  |  |  |  |
| None | 1785 (47.1) |  | 2184 (77.5) |  | 2901 (79.5) |  | 74.0 |  | 716 (26.3) |  | 354 (35.8) |  | 447 (37.4) |  | 52.8 |
| 1 | 1014 (26.8) |  | 444 (15.8) |  | 553 (15.2) |  | 49.6 |  | 1014 (37.3) |  | 444 (44.9) |  | 553 (46.3) |  | 49.6 |
| 2 | 691 (18.2) |  | 158 (5.6) |  | 162 (4.4) |  | 31.7 |  | 691 (25.4) |  | 158 (16.0) |  | 162 (13.6) |  | 31.7 |
| ≥3 | 300 (7.9) |  | 33 (1.2) |  | 33 (0.9) |  | 18.0 |  | 300 (11.0) |  | 33 (3.3) |  | 33 (2.8) |  | 18.0 |
| **Symptoms** **- no. (%)** |  |  |  |  |  |  |  |  |  |  |  |  |  |  |  |
| Fever | 869 (22.9) |  | 659 (23.4) |  | 641 (17.6) |  | 59.9 |  | 464 (17.1) |  | 185 (18.7) |  | 180 (15.1) |  | 44.0 |
| Cough | 1365 (36.0) |  | 1037 (36.8) |  | 1328 (36.4) |  | 63.4 |  | 907 (33.3) |  | 325 (32.9) |  | 394 (33.0) |  | 44.2 |
| Expectoration | 753 (19.9) |  | 473 (16.8) |  | 575 (15.8) |  | 58.2 |  | 537 (19.7) |  | 172 (17.4) |  | 197 (16.5) |  | 40.7 |
| Dizziness | 162 (4.3) |  | 105 (3.7) |  | 145 (4.0) |  | 60.7 |  | 96 (3.5) |  | 33 (3.3) |  | 31 (2.6) |  | 40.0 |
| Headache | 145 (3.8) |  | 145 (5.1) |  | 168 (4.6) |  | 68.3 |  | 84 (3.1) |  | 41 (4.1) |  | 38 (3.2) |  | 48.5 |
| Sore throat | 449 (11.8) |  | 472 (16.7) |  | 627 (17.2) |  | 71.0 |  | 276 (10.1) |  | 149 (15.1) |  | 153 (12.8) |  | 52.3 |
| Myalgia | 157 (4.1) |  | 180 (6.4) |  | 219 (6.0) |  | 71.8 |  | 79 (2.9) |  | 42 (4.2) |  | 51 (4.3) |  | 54.1 |
| Runny nose | 256 (6.8) |  | 264 (9.4) |  | 335 (9.2) |  | 70.1 |  | 131 (4.8) |  | 60 (6.1) |  | 78 (6.5) |  | 51.3 |
| Chest tightness | 80 (2.1) |  | 42 (1.5) |  | 35 (1.0) |  | 49.0 |  | 75 (2.8) |  | 20 (2.0) |  | 18 (1.5) |  | 33.6 |
| Chest pain | 16 (0.4) |  | 8 (0.3) |  | 12 (0.3) |  | 55.6 |  | 15 (0.6) |  | 4 (0.4) |  | 7 (0.6) |  | 42.3 |
| Shortness of breath | 77 (2.0) |  | 23 (0.8) |  | 20 (0.5) |  | 35.8 |  | 71 (2.6) |  | 13 (1.3) |  | 13 (1.1) |  | 26.8 |
| Diarrhea | 64 (1.7) |  | 43 (1.5) |  | 32 (0.9) |  | 54.0 |  | 52 (1.9) |  | 11 (1.1) |  | 8 (0.7) |  | 26.8 |
| **Admission diagnosis – no. (%)** |  |  |  |  |  |  |  |  |  |  |  |  |  |  |  |
| Asymptomatic | 707 (18.7) |  | 685 (24.3) |  | 938 (25.7) |  | 69.7 |  | 510 (18.7) |  | 257 (26.0) |  | 315 (26.4) |  | 52.9 |
| Mild | 2428 (64.1) |  | 2020 (71.7) |  | 2621 (71.8) |  | 65.7 |  | 1576 (57.9) |  | 644 (65.1) |  | 814 (68.1) |  | 48.1 |
| Moderate | 518 (13.7) |  | 94 (3.3) |  | 79 (2.2) |  | 25.0 |  | 500 (18.4) |  | 72 (7.3) |  | 57 (4.8) |  | 20.5 |
| Severe/critical | 137 (3.6) |  | 20 (0.7) |  | 11 (0.3) |  | 18.5 |  | 135 (5.0) |  | 16 (1.6) |  | 9 (0.8) |  | 15.6 |

^#^：Vaccination coverage includes both full vaccination and booster vaccination.

**Table S2 Inactivated vaccines prevented pneumonia in COVID-19 inpatients.**

| **Pneumonia** | **Analysis** | **Incomplete vaccination** | **Full vaccination** | **Booster vaccination** |
| --- | --- | --- | --- | --- |
| Full population | | | | |
|  | No. of events/ no. of patients at risk (%) | 655/3790(17.28) | 114/2819(4.04) | 90/3649(2.47) |
|  | Crude analysis- RR (95%CI) | Ref. | 0.234(0.191-0.284)^*^ | 0.143(0.114-0.177)^*^ |
|  | Multivariate^a^- RR (95%CI) | Ref. | 0.543(0.437-0.670)^*^ | 0.347(0.272-0.437)^*^ |
|  | CBPS IPTW- RR (95%CI) | Ref. | 0.580(0.526-0.638)^*^ | 0.398(0.357-0.444)^*^ |
| Full high-risk population | | | | |
|  | No. of events/ no. of patients at risk (%) | 635/2721(23.33) | 88/989(8.91) | 66/1195(5.53) |
|  | Crude analysis - RR (95%CI) | Ref. | 0.381(0.303-0.474)^*^ | 0.236(0.182-0.302)^*^ |
|  | Multivariate^a^ - RR (95%CI) | Ref. | 0.544(0.429-0.683)^*^ | 0.341(0.259-0.440)^*^ |
|  | CBPS IPTW - RR (95%CI) | Ref. | 0.585(0.528-0.648)^*^ | 0.399(0.355-0.447)^*^ |
| Aged <60 and with comorbidities | | | | |
|  | No. of events/ no. of patients at risk (%) | 27/217(12.44) | 12/210(5.71) | 8/290(2.76) |
|  | Crude analysis - RR (95%CI) | Ref. | 0.459(0.224-0.886)^*^ | 0.222(0.094-0.466)^*^ |
|  | Multivariate^a^ - RR (95%CI) | Ref. | 0.479(0.231-0.935)^*^ | 0.237(0.099-0.512)^*^ |
|  | CBPS IPTW - RR (95%CI) | Ref. | 0.471(0.321-0.679)^*^ | 0.266(0.165-0.414)^*^ |
| Aged ≥60 and without comorbidities | | | | |
|  | No. of events/ no. of patients at risk (%) | 145/716(20.25) | 25/354(7.06) | 17/447(3.80) |
|  | Crude analysis- RR (95%CI) | Ref. | 0.349(0.223-0.523)^*^ | 0.188(0.109-0.301)^*^ |
|  | Multivariate^a^- RR (95%CI) | Ref. | 0.471(0.295-0.723)^*^ | 0.252(0.145-0.414)^*^ |
|  | CBPS IPTW- RR (95%CI) | Ref. | 0.542(0.442-0.662)^*^ | 0.258(0.196-0.334)^*^ |
| Aged ≥60 and with comorbidities | | | | |
|  | No. of events/ no. of patients at risk (%) | 463/1788(25.90) | 51/424(12.0) | 41/456(8.99) |
|  | Crude analysis- RR (95%CI) | Ref. | 0.464(0.344-0.614)^*^ | 0.347(0.248-0.471)^*^ |
|  | Multivariate^a^- RR (95%CI) | Ref. | 0.623(0.458-0.831)^*^ | 0.464(0.330-0.635)^*^ |
|  | CBPS IPTW- RR (95%CI) | Ref. | 0.667(0.590-0.754)^*^ | 0.470(0.409-0.539)^*^ |
| Non-high risk population | | | | |
|  | No. of events/ no. of patients at risk (%) | 20/1069(1.87) | 26/1830(1.42) | 24/2454(0.98) |
|  | Crude analysis - RR (95%CI) | Ref. | 0.759(0.425-1.376) | 0.523(0.289-0.955)^*^ |
|  | Multivariate^a^ - RR (95%CI) | Ref. | 0.737(0.412-1.336) | 0.495(0.273-0.906)^*^ |
|  | CBPS IPTW - RR (95%CI) | Ref. | 0.730(0.542-0.978)^*^ | 0.490(0.348-0.681)^*^ |

CBPS IPTW, covariate-balancing propensity score inverse probability treatment weighting; RR, relative risk.

a: Age, sex and complications were adjusted.

*: statistical significant with p < 0.05.

In non-high risk population, booster vaccination reduced the rate ratio of pneumonia incidence, with statistical significance. However, the rate of pneumonia incidence was very low in non-high risk group, which indicates that the clinical effect of vaccination might be negligible in this population.

**Table S3 Inactivated vaccines prevented severe outcomes in COVID-19 inpatients.**

| **Severe outcomes** | **Analysis** | **Incomplete vaccination** | **Full vaccination** | **Booster vaccination** |
| --- | --- | --- | --- | --- |
| Full population | | | | |
|  | No. of events/ no. of patients at risk (%) | 287/3790(7.57) | 37/2819(1.31) | 20/3649(0.55) |
|  | Crude analysis- RR (95%CI) | Ref. | 0.173(0.121-0.241)^*^ | 0.072(0.044-0.111)^*^ |
|  | Multivariate^a^- RR (95%CI) | Ref. | 0.517(0.353-0.738)^*^ | 0.229(0.138-0.359)^*^ |
|  | CBPS IPTW- RR (95%CI) | Ref. | 0.613(0.527-0.711)^*^ | 0.376(0.314-0.447)^*^ |
| Full high-risk population | | | | |
|  | No. of events/ no. of patients at risk (%) | 285/2721(10.47) | 32/989(3.24) | 17/1195(1.42) |
|  | Crude analysis- RR (95%CI) | Ref. | 0.309(0.210-0.438)^*^ | 0.136(0.080-0.214)^*^ |
|  | Multivariate^a^- RR (95%CI) | Ref. | 0.524(0.351-0.755)^*^ | 0.233(0.135-0.373)^*^ |
|  | CBPS IPTW- RR (95%CI) | Ref. | 0.613(0.524-0.714)^*^ | 0.384(0.320-0.458)^*^ |
| Aged <60 and with comorbidities | | | | |
|  | No. of events/ no. of patients at risk (%) | 9/217(4.15) | 3/210(1.43) | 3/290(1.03) |
|  | Crude analysis- RR (95%CI) | Ref. | 0.343(0.076-1.149) | 0.248(0.055-0.830)^*^ |
|  | Multivariate^a^- RR (95%CI) | Ref. | 0.376(0.082-1.288) | 0.292(0.063-1.040) |
|  | CBPS IPTW- RR (95%CI) | Ref. | 0.359(0.166-0.716)^*^ | 0.253(0.102-0.547)^*^ |
| Aged ≥60 and without comorbidities | | | | |
|  | No. of events/ no. of patients at risk (%) | 54/716(7.54) | 8/354(2.26) | 2/447(0.45) |
|  | Crude analysis- RR (95%CI) | Ref. | 0.300(0.132-0.593)^*^ | 0.059(0.010-0.190)^*^ |
|  | Multivariate^a^- RR (95%CI) | Ref. | 0.554(0.235-1.159) | 0.107(0.017-0.359)^*^ |
|  | CBPS IPTW- RR (95%CI) | Ref. | 0.584(0.412-0.817)^*^ | 0.271(0.169-0.418)^*^ |
| Aged ≥60 and with comorbidities | | | | |
|  | No. of events/ no. of patients at risk (%) | 222/1788(12.42) | 21/424(4.95) | 12/456(2.63) |
|  | Crude analysis- RR (95%CI) | Ref. | 0.399(0.247-0.608)^*^ | 0.212(0.112-0.362)^*^ |
|  | Multivariate^a^- RR (95%CI) | Ref. | 0.613(0.376-0.948)^*^ | 0.324(0.170-0.561)^*^ |
|  | CBPS IPTW- RR (95%CI) | Ref. | 0.695(0.581-0.831)^*^ | 0.432(0.350-0.531)^*^ |
| Non-high risk population | | | | |
|  | No. of events/ no. of patients at risk (%) | 2/1069(0.19) | 5/1830(0.27) | 3/2454(0.12) |
|  | Crude analysis- RR (95%CI) | Ref. | 1.460(0.315-10.197) | 0.653(0.108-4.961) |
|  | Multivariate^a^- RR (95%CI) | Ref. | 1.392(0.300-9.731) | 0.599(0.099-4.563) |
|  | CBPS IPTW- RR (95%CI) | Ref. | 1.275(0.590-2.827) | 0.535(0.187-1.387) |

CBPS IPTW, covariate-balancing propensity score inverse probability treatment weighting; RR, relative risk.

a: Age, sex and complications were adjusted.

*: statistical significant with p < 0.0

**Table S4** **Baseline and clinical characteristics of COVID-19 inpatients who had disease progression.**

| **Characteristics** | **Progress 1**  **(N=119)** | **Progress 2**  **(N=24)** | **Progress 3**  **(N=56)** | **Total**  **(N=199)** |
| --- | --- | --- | --- | --- |
| Median age (IQR) - yr | 75 (68-85) | 81 (75-88) | 83 (76-89) | 78 (69-87) |
| Age group - no. (%) |  |  |  |  |
| <40 yr | 2 (1.7) | 0 (0.0) | 1 (1.8) | 3 (1.5) |
| 40 to 49 yr | 4 (3.4) | 0 (0.0) | 0 (0.0) | 4 (2.0) |
| 50 to 59 yr | 11 (9.2) | 0 (0.0) | 4 (7.1) | 15 (7.5) |
| 60 to 69 yr | 22 (18.5) | 0 (0.0) | 6 (10.7) | 28 (14.1) |
| 70 to 79 yr | 37 (31.1) | 8 (33.3) | 8 (14.3) | 53 (26.6) |
| ≥80 yr | 43 (36.1) | 16 (66.7) | 37 (66.1) | 96 (48.2) |
| Gender - no. (%) |  |  |  |  |
| Female | 75 (63.0) | 12 (50.0) | 26 (46.4) | 113 (56.8) |
| Male | 44 (37.0) | 12 (50.0) | 30 (53.6) | 86 (43.2) |
| Number of comorbidities - no. (%) |  |  |  |  |
| None | 30 (25.2) | 3 (12.5) | 14 (25.0) | 47 (23.6) |
| 1 | 46 (38.7) | 12 (50.0) | 14 (25.0) | 72 (36.2) |
| 2 | 29 (24.4) | 6 (25.0) | 19 (33.9) | 54 (27.1) |
| ≥3 | 14 (11.8) | 3 (12.5) | 9 (16.1) | 26 (13.1) |
| Vaccination status - no. (%) |  |  |  |  |
| Incomplete vaccination | 79 (66.4) | 19 (79.2) | 47 (83.9) | 145 (72.9) |
| Full vaccination | 15 (12.6) | 3 (12.5) | 6 (10.7) | 24 (12.1) |
| Booster vaccination | 25 (21.0) | 2 (8.3) | 3 (5.4) | 30 (15.1) |

Progress 1: inpatients progressed to moderate infection from asymptomatic/mild infection during hospitalization;

Progress 2: inpatients progressed to severe/critical infection from asymptomatic/mild infection during hospitalization;

Progress 3: inpatients progressed to severe/critical infection from moderate infection during hospitalization.

**Table S5 Inactivated vaccines prevented disease progression in COVID-19 inpatients.**

| **Progress** | **Analysis** | **Incomplete vaccination** | **Full vaccination** | **Booster vaccination** |
| --- | --- | --- | --- | --- |
| Full population | | | | |
|  | No. of events/ no. of patients at risk (%) | 145/3653(3.97) | 24/2799(0.86) | 30/3638(0.82) |
|  | Crude analysis- RR (95%CI) | Ref. | 0.216(0.137-0.326)^*^ | 0.208(0.138-0.303)^*^ |
|  | Multivariate^a^- RR (95%CI) | Ref. | 0.549(0.339-0.854)^*^ | 0.577(0.370-0.874)^*^ |
|  | CBPS IPTW- RR (95%CI) | Ref. | 0.507(0.409-0.624)^*^ | 0.562(0.457-0.688)^*^ |
| Full high-risk population | | | | |
|  | No. of events/ no. of patients at risk (%) | 142/2586(5.49) | 21/973(2.16) | 24/1186(2.02) |
|  | Crude analysis - RR (95%CI) | Ref. | 0.393(0.242-0.607)^*^ | 0.369(0.233-0.557)^*^ |
|  | Multivariate^a^ - RR (95%CI) | Ref. | 0.573(0.346-0.905)^*^ | 0.560(0.347-0.871)^*^ |
|  | CBPS IPTW - RR (95%CI) | Ref. | 0.506(0.405-0.628)^*^ | 0.548(0.442-0.676)^*^ |
| Aged <60 and with comorbidities | | | | |
|  | No. of events/ no. of patients at risk (%) | 6/212(2.83) | 1/208(0.48) | 3/288(1.04) |
|  | Crude analysis - RR (95%CI) | Ref. | 0.170(0.009-0.994)^*^ | 0.368(0.078-1.395) |
|  | Multivariate^a^ - RR (95%CI) | Ref. | 0.173(0.009-1.013) | 0.382(0.081-1.453) |
|  | CBPS IPTW - RR (95%CI) | Ref. | 0.168(0.043-0.471)^*^ | 0.380(0.154-0.842)^*^ |
| Aged ≥60 and without comorbidities | | | | |
|  | No. of events/ no. of patients at risk (%) | 25/690(3.62) | 5/350(1.43) | 5/447(1.12) |
|  | Crude analysis- RR (95%CI) | Ref. | 0.394(0.133-0.947)^*^ | 0.309(0.104-0.742)^*^ |
|  | Multivariate^a^- RR (95%CI) | Ref. | 0.394(0.133-0.947)^*^ | 0.309(0.104-0.745)^*^ |
|  | CBPS IPTW- RR (95%CI) | Ref. | 0.390(0.231-0.634)^*^ | 0.335(0.191-0.558)^*^ |
| Aged ≥60 and with comorbidities | | | | |
|  | No. of events/ no. of patients at risk (%) | 111/1684(6.59) | 15/414(3.62) | 16/449(3.56) |
|  | Crude analysis- RR (95%CI) | Ref. | 0.550(0.308-0.912)^*^ | 0.541(0.308-0.885)^*^ |
|  | Multivariate^a^- RR (95%CI) | Ref. | 0.548(0.307-0.909)^*^ | 0.544(0.310-0.890)^*^ |
|  | CBPS IPTW- RR (95%CI) | Ref. | 0.547(0.422-0.703)^*^ | 0.557(0.431-0.716)^*^ |
| Non-high risk population | | | | |
|  | No. of events/ no. of patients at risk (%) | 3/1067(0.28) | 3/1826(0.16) | 6/2452(0.24) |
|  | Crude analysis - RR (95%CI) | Ref. | 0.584(0.108-3.157) | 0.870(0.230-4.124) |
|  | Multivariate^a^ - RR (95%CI) | Ref. | 0.601(0.111-3.251) | 0.917(0.241-4.359) |
|  | CBPS IPTW - RR (95%CI) | Ref. | 0.541(0.225-1.211) | 0.838(0.399-1.734)  . |

CBPS IPTW, covariate-balancing propensity score inverse probability treatment weighting; RR, relative risk.

a: Age, sex and complications were adjusted.

*: statistical significant with p < 0.05.

**Table S6 Inactivated vaccines reduced viral shedding time of SARS-CoV-2.**

| **Viral shedding time** | **Analysis** | **Incomplete vaccination** | **Full vaccination** | **Booster vaccination** |
| --- | --- | --- | --- | --- |
| Full population | | | | |
|  | Median time to recovery (IQR) - days | 13(9-17) | 10(7-14) | 10(7-13) |
|  | Crude analysis- HR (95%CI) | Ref. | 1.589(1.513-1.669)^*^ | 1.727(1.649-1.808)^*^ |
|  | Multivariate^a^- HR (95%CI) | Ref. | 1.248(1.184-1.316)^*^ | 1.329(1.265-1.397)^*^ |
|  | CBPS IPTW- HR (95%CI) | Ref. | 1.203(1.126-1.285)^*^ | 1.207(1.120-1.300)^*^ |
| Full high-risk population | | | | |
|  | Median time to recovery (IQR) | 14(10-18) | 12(8-16) | 11(8-15) |
|  | Crude analysis- HR(95%CI) | Ref. | 1.356(1.261-1.459)^*^ | 1.461(1.364-1.564)^*^ |
|  | Multivariate^a^- HR(95%CI) | Ref. | 1.210(1.122-1.306)^*^ | 1.290(1.200-1.387)^*^ |
|  | CBPS IPTW- HR(95%CI) | Ref. | 1.258(1.157-1.366)^*^ | 1.314(1.216-1.421)^*^ |
| Aged <60 and with comorbidities | | | | |
|  | Median time to recovery (IQR) | 13(11-16) | 11(8-14) | 10(7-13) |
|  | Crude analysis- HR(95%CI) | Ref. | 1.429(1.182-1.729)^*^ | 1.542(1.292-1.840)^*^ |
|  | Multivariate^a^- HR(95%CI) | Ref. | 1.437(1.187-1.738)^*^ | 1.555(1.303-1.856)^*^ |
|  | CBPS IPTW- HR(95%CI) | Ref. | 1.447(1.211-1.730)^*^ | 1.540(1.287-1.843)^*^ |
| Aged ≥60 and without comorbidities | | | | |
|  | Median time to recovery (IQR) | 13(9-16) | 11(7-15) | 11(8-15) |
|  | Crude analysis- HR(95%CI) | Ref. | 1.331(1.171-1.512)^*^ | 1.365(1.212-1.537)^*^ |
|  | Multivariate^a^- HR(95%CI) | Ref. | 1.331(1.171-1.512)^*^ | 1.361(1.208-1.532)^*^ |
|  | CBPS IPTW- HR(95%CI) | Ref. | 1.325(1.167-1.505)^*^ | 1.330(1.175-1.506)^*^ |
| Aged ≥60 and with comorbidities | | | | |
|  | Median time to recovery (IQR) | 14(10-18) | 13(8-16) | 12(8-16) |
|  | Crude analysis- HR(95%CI) | Ref. | 1.235(1.111-1.374)^*^ | 1.374(1.239-1.523)^*^ |
|  | Multivariate^a^- HR(95%CI) | Ref. | 1.234(1.110-1.373)^*^ | 1.372(1.237-1.522)^*^ |
|  | CBPS IPTW- HR(95%CI) | Ref. | 1.236(1.107-1.380)^*^ | 1.353(1.217-1.504)^*^ |
| Non-high risk population | | | | |
|  | Median time to recovery (IQR) | 11(9-14) | 10(7-12) | 9(7-12) |
|  | Crude analysis- HR(95%CI) | Ref. | 1.287(1.193-1.388)^*^ | 1.388(1.291-1.491)^*^ |
|  | Multivariate^a^- HR(95%CI) | Ref. | 1.286(1.192-1.386)^*^ | 1.367(1.272-1.469)^*^ |
|  | CBPS IPTW- HR(95%CI) | Ref. | 1.283(1.195-1.377)^*^ | 1.370(1.280-1.467)^*^ |

IQR, interquartile range; CBPS IPTW, covariate-balancing propensity score inverse probability treatment weighting; HR, hazard ratio.

a: Age, sex and complications were adjusted.

*: statistical significant with p < 0.05.

**Table S7 Baseline and clinical characteristics of West Bund population.**

|  | **Full population** | | | | **High-risk population** | | | |
| --- | --- | --- | --- | --- | --- | --- | --- | --- |
|  | **Incomplete vaccination**  **(N=1461)** | **Full vaccination**  **(N=1243)** | **Booster vaccination**  **(N=2186)** | **Total**  **(N=4890)** | **Incomplete vaccination**  **(N=1230)** | **Full vaccination**  **(N=437)** | **Booster vaccination**  **(N=682)** | **Total**  **(N=2349)** |
| **Characteristics** |  |  |  |  |  |  |  |  |
| Median age (IQR) - yr | 73 (63, 85) | 48 (32, 62) | 50 (39, 59) | 55 (40, 70) | 77 (68, 87) | 66 (61, 72) | 65 (60, 71) | 70 (63, 81) |
| Age group - no. (%) |  |  |  |  |  |  |  |  |
| <40 yr | 143 (9.8) | 484 (38.9) | 579 (26.5) | 1206 (24.7) | 15 (1.2) | 12 (2.7) | 15 (2.2) | 42 (1.8) |
| 40 to 49 yr | 60 (4.1) | 174 (14.0) | 466 (21.3) | 700 (14.3) | 17 (1.4) | 18 (4.1) | 39 (5.7) | 74 (3.2) |
| 50 to 59 yr | 107 (7.3) | 233 (18.7) | 613 (28.0) | 953 (19.5) | 47 (3.8) | 55 (12.6) | 100 (14.7) | 202 (8.6) |
| 60 to 69 yr | 286 (19.6) | 201 (16.2) | 320 (14.6) | 807 (16.5) | 286 (23.3) | 201 (46.0) | 320 (46.9) | 807 (34.4) |
| 70 to 79 yr | 318 (21.8) | 108 (8.7) | 154 (7.0) | 580 (11.9) | 318 (25.9) | 108 (24.7) | 154 (22.6) | 580 (24.7) |
| ≥80 yr | 547 (37.4) | 43 (3.5) | 54 (2.5) | 644 (13.2) | 547 (44.5) | 43 (9.8) | 54 (7.9) | 644 (27.4) |
| Male - no. (%) | 693 (47.4) | 686 (55.2) | 1216 (55.6) | 2595 (53.1) | 557 (45.3) | 205 (46.9) | 371 (54.4) | 1133 (48.2) |
| Number of comorbidities - no. (%) |  |  |  |  |  |  |  |  |
| None | 542 (37.1) | 970 (78.0) | 1752 (80.1) | 3264 (66.7) | 311 (25.3) | 164 (37.5) | 248 (36.4) | 723 (30.8) |
| 1 | 472 (32.3) | 190 (15.3) | 324 (14.8) | 986 (20.2) | 472 (38.4) | 190 (43.5) | 324 (47.5) | 986 (42.0) |
| 2 | 319 (21.8) | 71 (5.7) | 87 (4.0) | 477 (9.8) | 319 (25.9) | 71 (16.2) | 87 (12.8) | 477 (20.3) |
| ≥3 | 128 (8.8) | 12 (1.0) | 23 (1.1) | 163 (3.3) | 128 (10.4) | 12 (2.7) | 23 (3.4) | 163 (6.9) |
| **Symptoms** |  |  |  |  |  |  |  |  |
| Fever - no. (%) | 226 (15.5) | 214 (17.2) | 305 (14.0) | 745 (15.2) | 189 (15.4) | 71 (16.2) | 96 (14.1) | 356 (15.2) |
| Cough - no. (%) | 434 (29.7) | 334 (26.9) | 686 (31.4) | 1454 (29.7) | 372 (30.2) | 116 (26.5) | 227 (33.3) | 715 (30.4) |
| Expectoration - no. (%) | 226 (15.5) | 107 (8.6) | 191 (8.7) | 524 (10.7) | 204 (16.6) | 46 (10.5) | 95 (13.9) | 345 (14.7) |
| Dizziness - no. (%) | 25 (1.7) | 21 (1.7) | 36 (1.6) | 82 (1.7) | 23 (1.9) | 10 (2.3) | 10 (1.5) | 43 (1.8) |
| Headache - no. (%) | 33 (2.3) | 32 (2.6) | 56 (2.6) | 121 (2.5) | 27 (2.2) | 10 (2.3) | 14 (2.1) | 51 (2.2) |
| Sore throat - no. (%) | 129 (8.8) | 151 (12.1) | 321 (14.7) | 601 (12.3) | 103 (8.4) | 49 (11.2) | 75 (11.0) | 227 (9.7) |
| Myalgia - no. (%) | 37 (2.5) | 63 (5.1) | 91 (4.2) | 191 (3.9) | 23 (1.9) | 20 (4.6) | 25 (3.7) | 68 (2.9) |
| Runny nose - no. (%) | 86 (5.9) | 87 (7.0) | 166 (7.6) | 339 (6.9) | 67 (5.4) | 28 (6.4) | 46 (6.7) | 141 (6.0) |
| Chest tightness - no. (%) | 23 (1.6) | 9 (0.7) | 10 (0.5) | 42 (0.9) | 23 (1.9) | 4 (0.9) | 5 (0.7) | 32 (1.4) |
| Chest pain - no. (%) | 5 (0.3) | 0 (0.0) | 3 (0.1) | 8 (0.2) | 5 (0.4) | 0 (0.0) | 2 (0.3) | 7 (0.3) |
| Shortness of breath - no. (%) | 27 (1.8) | 6 (0.5) | 8 (0.4) | 41 (0.8) | 25 (2.0) | 4 (0.9) | 4 (0.6) | 33 (1.4) |
| Diarrhea - no. (%) | 19 (1.3) | 22 (1.8) | 19 (0.9) | 60 (1.2) | 17 (1.4) | 4 (0.9) | 5 (0.7) | 26 (1.1) |
| **Admission diagnosis – no. (%)** |  |  |  |  |  |  |  |  |
| Asymptomatic | 278 (19.0) | 323 (26.0) | 575 (26.3) | 1176 (24.0) | 218 (17.7) | 99 (22.7) | 155 (22.7) | 472 (20.1) |
| Mild | 974 (66.7) | 890 (71.6) | 1578 (72.2) | 3442 (70.4) | 805 (65.4) | 314 (71.9) | 498 (73.0) | 1617 (68.8) |
| Moderate | 159 (10.9) | 25 (2.0) | 27 (1.2) | 211 (4.3) | 158 (12.8) | 19 (4.3) | 24 (3.5) | 201 (8.6) |
| Severe/critical | 50 (3.4) | 5 (0.4) | 6 (0.3) | 61 (1.2) | 49 (4.0) | 5 (1.1) | 5 (0.7) | 59 (2.5) |

**Table S8 Baseline and clinical characteristics of East Bund population.**

|  | **Full population** | | | | **High-risk population** | | | |
| --- | --- | --- | --- | --- | --- | --- | --- | --- |
|  | **Incomplete vaccination**  **(N=2329)** | **Full vaccination**  **(N=1576)** | **Booster vaccination**  **(N=1463)** | **Total**  **(N=5368)** | **Incomplete vaccination**  **(N=1491)** | **Full vaccination**  **(N=552)** | **Booster vaccination**  **(N=513)** | **Total**  **(N=2556)** |
| **Characteristics** |  |  |  |  |  |  |  |  |
| Median age (IQR) - yr | 65 (32, 80) | 41 (31, 62) | 49 (35, 60) | 52 (33, 70) | 75 (67, 85) | 67 (60, 73) | 66 (58, 71) | 71 (64, 80) |
| Age group - no. (%) |  |  |  |  |  |  |  |  |
| <40 yr | 742 (31.9) | 738 (46.8) | 505 (34.5) | 1985 (37.0) | 33 (2.2) | 25 (4.5) | 23 (4.5) | 81 (3.2) |
| 40 to 49 yr | 94 (4.0) | 188 (11.9) | 240 (16.4) | 522 (9.7) | 29 (1.9) | 28 (5.1) | 31 (6.0) | 88 (3.4) |
| 50 to 59 yr | 140 (6.0) | 224 (14.2) | 343 (23.4) | 707 (13.2) | 76 (5.1) | 73 (13.2) | 84 (16.4) | 233 (9.1) |
| 60 to 69 yr | 356 (15.3) | 216 (13.7) | 213 (14.6) | 785 (14.6) | 356 (23.9) | 216 (39.1) | 213 (41.5) | 785 (30.7) |
| 70 to 79 yr | 411 (17.6) | 158 (10.0) | 129 (8.8) | 698 (13.0) | 411 (27.6) | 158 (28.6) | 129 (25.1) | 698 (27.3) |
| ≥80 yr | 586 (25.2) | 52 (3.3) | 33 (2.3) | 671 (12.5) | 586 (39.3) | 52 (9.4) | 33 (6.4) | 671 (26.3) |
| Male - no. (%) | 1027 (44.1) | 630 (40.0) | 674 (46.1) | 2331 (43.4) | 717 (48.1) | 256 (46.4) | 269 (52.4) | 1242 (48.6) |
| Number of comorbidities - no. (%) |  |  |  |  |  |  |  |  |
| None | 1243 (53.4) | 1214 (77.0) | 1149 (78.5) | 3606 (67.2) | 405 (27.2) | 190 (34.4) | 199 (38.8) | 794 (31.1) |
| 1 | 542 (23.3) | 254 (16.1) | 229 (15.7) | 1025 (19.1) | 542 (36.4) | 254 (46.0) | 229 (44.6) | 1025 (40.1) |
| 2 | 372 (16.0) | 87 (5.5) | 75 (5.1) | 534 (9.9) | 372 (24.9) | 87 (15.8) | 75 (14.6) | 534 (20.9) |
| ≥3 | 172 (7.4) | 21 (1.3) | 10 (0.7) | 203 (3.8) | 172 (11.5) | 21 (3.8) | 10 (1.9) | 203 (7.9) |
| **Symptoms** |  |  |  |  |  |  |  |  |
| Fever - no. (%) | 643 (27.6) | 445 (28.2) | 336 (23.0) | 1424 (26.5) | 275 (18.4) | 114 (20.7) | 84 (16.4) | 473 (18.5) |
| Cough - no. (%) | 931 (40.0) | 703 (44.6) | 642 (43.9) | 2276 (42.4) | 535 (35.9) | 209 (37.9) | 167 (32.6) | 911 (35.6) |
| Expectoration - no. (%) | 527 (22.6) | 366 (23.2) | 384 (26.2) | 1277 (23.8) | 333 (22.3) | 126 (22.8) | 102 (19.9) | 561 (21.9) |
| Dizziness - no. (%) | 137 (5.9) | 84 (5.3) | 109 (7.5) | 330 (6.1) | 73 (4.9) | 23 (4.2) | 21 (4.1) | 117 (4.6) |
| Headache - no. (%) | 112 (4.8) | 113 (7.2) | 112 (7.7) | 337 (6.3) | 57 (3.8) | 31 (5.6) | 24 (4.7) | 112 (4.4) |
| Sore throat - no. (%) | 320 (13.7) | 321 (20.4) | 306 (20.9) | 947 (17.6) | 173 (11.6) | 100 (18.1) | 78 (15.2) | 351 (13.7) |
| Myalgia - no. (%) | 120 (5.2) | 117 (7.4) | 128 (8.7) | 365 (6.8) | 56 (3.8) | 22 (4.0) | 26 (5.1) | 104 (4.1) |
| Runny nose - no. (%) | 170 (7.3) | 177 (11.2) | 169 (11.6) | 516 (9.6) | 64 (4.3) | 32 (5.8) | 32 (6.2) | 128 (5.0) |
| Chest tightness - no. (%) | 57 (2.4) | 33 (2.1) | 25 (1.7) | 115 (2.1) | 52 (3.5) | 16 (2.9) | 13 (2.5) | 81 (3.2) |
| Chest pain - no. (%) | 11 (0.5) | 8 (0.5) | 9 (0.6) | 28 (0.5) | 10 (0.7) | 4 (0.7) | 5 (1.0) | 19 (0.7) |
| Shortness of breath - no. (%) | 50 (2.1) | 17 (1.1) | 12 (0.8) | 79 (1.5) | 46 (3.1) | 9 (1.6) | 9 (1.8) | 64 (2.5) |
| Diarrhea - no. (%) | 45 (1.9) | 21 (1.3) | 13 (0.9) | 79 (1.5) | 35 (2.3) | 7 (1.3) | 3 (0.6) | 45 (1.8) |
| **Admission diagnosis -no. (%)** |  |  |  |  |  |  |  |  |
| Asymptomatic | 429 (18.4) | 362 (23.0) | 363 (24.8) | 1154 (21.5) | 292 (19.6) | 158 (28.6) | 160 (31.2) | 610 (23.9) |
| Mild | 1454 (62.4) | 1130 (71.7) | 1043 (71.3) | 3627 (67.6) | 771 (51.7) | 330 (59.8) | 316 (61.6) | 1417 (55.4) |
| Moderate | 359 (15.4) | 69 (4.4) | 52 (3.6) | 480 (8.9) | 342 (22.9) | 53 (9.6) | 33 (6.4) | 428 (16.7) |
| Severe/critical | 87 (3.7) | 15 (1.0) | 5 (0.3) | 107 (2.0) | 86 (5.8) | 11 (2.0) | 4 (0.8) | 101 (4.0) |

**Table S9 Effectiveness of inactivated vaccines in preventing pneumonia in COVID-19 inpatients validated in West Bund population and East Bund population.**

| **Pneumonia** | **Analysis** | **Incomplete vaccination** | **Full vaccination** | **Booster vaccination** |
| --- | --- | --- | --- | --- |
| **West Bund population** | | | | |
| Full population | | | | |
|  | No. of events/ no. of patients at risk (%) | 209/1461(14.31) | 30/1243(2.41) | 33/2186(1.51) |
|  | Crude analysis- RR (95%CI) | Ref. | 0.169(0.113-0.163)^*^ | 0.105(0.072-0.150)^*^ |
|  | Multivariate^a^- RR (95%CI) | Ref. | 0.442(0.291-0.649)^*^ | 0.304(0.203-0.444)^*^ |
|  | CBPS IPTW- RR (95%CI) | Ref. | 0.400(0.332-0.479)^*^ | 0.335(0.275-0.405)^*^ |
| High-risk population | | | | |
|  | No. of events/ no. of patients at risk (%) | 207/1230(16.83) | 24/437(5.49) | 29/682(4.25) |
|  | Crude analysis - RR (95%CI) | Ref. | 0.326(0.208-0.487)^*^ | 0.252(0.168-0.366)^*^ |
|  | Multivariate^a^ - RR (95%CI) | Ref. | 0.415(0.264-0.625)^*^ | 0.326(0.214-0.477)^*^ |
|  | CBPS IPTW - RR (95%CI) | Ref. | 0.400(0.329-0.484)^*^ | 0.356(0.290-0.434)^*^ |
| Non-high risk population | | | | |
|  | No. of events/ no. of patients at risk (%) | 2/231(0.87) | 6/806(0.74) | 4/1504(0.27) |
|  | Crude analysis - RR (95%CI) | Ref. | 0.860(0.198-5.868) | 0.307(0.060-2.216) |
|  | Multivariate^a^ - RR (95%CI) | Ref. | 0.862(0.198-5.885) | 0.304(0.059-2.196) |
|  | CBPS IPTW - RR (95%CI) | Ref. | 0.863(0.463-1.592) | 0.304(0.118-0.685)^*^ |
| **East Bund population** | | | | |
| Full population | | | | |
|  | No. of events/ no. of patients at risk (%) | 446/2329(19.15) | 84/1576(5.33) | 57/1463(3.90) |
|  | Crude analysis- RR (95%CI) | Ref. | 0.278(0.219-0.349)^*^ | 0.203(0.153-0.265)^*^ |
|  | Multivariate^a^- RR (95%CI) | Ref. | 0.580(0.450-0.739)^*^ | 0.431(0.320-0.572)^*^ |
|  | CBPS IPTW- RR (95%CI) | Ref. | 0.615(0.546-0.693)^*^ | 0.426(0.372-0.486)^*^ |
| High-risk population | | | | |
|  | No. of events/ no. of patients at risk (%) | 428/1491(28.70) | 64/552(11.6) | 37/513(7.21) |
|  | Crude analysis- RR (95%CI) | Ref. | 0.404(0.308-0.521)^*^ | 0.251(0.177-0.346)^*^ |
|  | Multivariate^a^- RR (95%CI) | Ref. | 0.554(0.418-0.723)^*^ | 0.349(0.243-0.488)^*^ |
|  | CBPS IPTW- RR (95%CI) | Ref. | 0.604(0.532-0.684)^*^ | 0.390(0.337-0.450)^*^ |
| Non-high risk population | | | | |
|  | No. of events/ no. of patients at risk (%) | 18/838(2.15) | 20/1024(1.95) | 20/950(2.11) |
|  | Crude analysis - RR (95%CI) | Ref. | 0.909(0.480-1.734) | 0.980(0.517-1.869) |
|  | Multivariate^a^ - RR (95%CI) | Ref. | 0.914(0.482-1.743) | 0.924(0.487-1.764) |
|  | CBPS IPTW - RR (95%CI) | Ref. | 0.913(0.635-1.311) | 0.921(0.640-1.321) |

CBPS IPTW, covariate-balancing propensity score inverse probability treatment weighting; RR, relative risk.

a: Age, sex and complications were adjusted.

*: statistical significant with p < 0.05.

**Table S10 Effectiveness of inactivated vaccines in preventing severe outcomes in COVID-19 inpatients validated in West Bund population and East Bund population.**

| **Severe outcomes** | **Analysis** | **Incomplete vaccination** | **Full vaccination** | **Booster vaccination** |
| --- | --- | --- | --- | --- |
| **West Bund population** | | | | |
| Full population | | | | |
|  | No. of events/ no. of patients at risk (%) | 114/1461(7.80) | 11/1243(0.88) | 11/2186(0.50) |
|  | Crude analysis- RR (95%CI) | Ref. | 0.113(0.057-0.201)^*^ | 0.064(0.033-0.114)^*^ |
|  | Multivariate^a^- RR (95%CI) | Ref. | 0.456(0.222-0.850)^*^ | 0.286(0.138-0.539)^*^ |
|  | CBPS IPTW- RR (95%CI) | Ref. | 0.388(0.299-0.500)^*^ | 0.525(0.415-0.661)^*^ |
| High-risk population | | | | |
|  | No. of events/ no. of patients at risk (%) | 113/1230(9.19) | 11/437(2.52) | 10/682(1.47) |
|  | Crude analysis- RR (95%CI) | Ref. | 0.274(0.139-0.485)^*^ | 0.160(0.078-0.289)^*^ |
|  | Multivariate^a^- RR (95%CI) | Ref. | 0.550(0.273-1.006) | 0.321(0.154-0.604)^*^ |
|  | CBPS IPTW- RR (95%CI) | Ref. | 0.464(0354-0.603)^*^ | 0.567(0.441-0.725)^*^ |
| Non-high risk population | | | | |
|  | No. of events/ no. of patients at risk (%) | 1/231(0.43) | 0/806(0.00) | 1/1504(0.07) |
|  | Crude analysis- RR (95%CI) | Ref. | / | 0.153(0.016-1.519) |
|  | Multivariate^a^- RR (95%CI) | Ref. | / | 0.161(0.029-0.900)^*^ |
|  | CBPS IPTW- RR (95%CI) | Ref. | / | 0.116(0.021-0.653)^*^ |
| **East Bund population** | | | | |
| Full population | | | | |
|  | No. of events/ no. of patients at risk (%) | 173/2329(7.43) | 26/1576(1.65) | 9/1463(0.62) |
|  | Crude analysis- RR (95%CI) | Ref. | 0.222(0.144-0.329)^*^ | 0.083(0.039-0.152)^*^ |
|  | Multivariate^a^- RR (95%CI) | Ref. | 0.569(0.359-0.867)^*^ | 0.219(0.102-0.412)^*^ |
|  | CBPS IPTW- RR (95%CI) | Ref. | 0.736(0.609-0.888)^*^ | 0.135(0.093-0.190)^*^ |
| High-risk population | | | | |
|  | No. of events/ no. of patients at risk (%) | 172/1491(11.54) | 21/552(3.80) | 7/513(1.36) |
|  | Crude analysis- RR (95%CI) | Ref. | 0.330(0.204-0.506)^*^ | 0.118(0.050-0.233)^*^ |
|  | Multivariate^a^- RR (95%CI) | Ref. | 0.511(0.310-0.802)^*^ | 0.187(0.078-0.375)^*^ |
|  | CBPS IPTW- RR (95%CI) | Ref. | 0.719(0.592-0.871)^*^ | 0.118(0.079-0.170)^*^ |
| Non-high risk population | | | | |
|  | No. of events/ no. of patients at risk (%) | 1/838(0.12) | 5/1024(0.49) | 2/950(0.21) |
|  | Crude analysis- RR (95%CI) | Ref. | 4.092(0.660-78.395) | 1.764(0.169,37.964) |
|  | Multivariate^a^- RR (95%CI) | Ref. | 4.112(0.663-78.783) | 1.666(1.593-35.870) |
|  | CBPS IPTW- RR (95%CI) | Ref. | 3.921(1.346-15.120)^*^ | 1.531(0.405-6.610) |

CBPS IPTW, covariate-balancing propensity score inverse probability treatment weighting; RR, relative risk.

a: Age, sex and complications were adjusted.

*: statistical significant with p < 0.05.

**Table S11 Effectiveness of inactivated vaccines in preventing disease progression in COVID-19 inpatients validated in West Bund population and East Bund population.**

| **Disease progression**  **Progress** | **Analysis** | **Incomplete vaccination** | **Full vaccination** | **Booster vaccination** |
| --- | --- | --- | --- | --- |
| **West Bund population** | | | | |
| Full population | | | | |
|  | No. of events/ no. of patients at risk (%) | 97/1411(6.87) | 15/1238(1.21) | 22/2180(1.01) |
|  | Crude analysis- RR (95%CI) | Ref. | 0.176(0.098-0.294)^*^ | 0.147(0.090-0.228)^*^ |
|  | Multivariate^a^- RR (95%CI) | Ref. | 0.577(0.311-1.002) | 0.543(0.317-0.897)^*^ |
|  | CBPS IPTW- RR (95%CI) | Ref. | 0.510(0.399-0.648)^*^ | 0.461(0.358-0.590)^*^ |
| High-risk population | | | | |
|  | No. of events/ no. of patients at risk (%) | 95/1181(804) | 14/432(3.24) | 20/677(2.95) |
|  | Crude analysis - RR (95%CI) | Ref. | 0.402(0.220-0.682)^*^ | 0.367(0.220-0.581)^*^ |
|  | Multivariate^a^ - RR (95%CI) | Ref. | 0.641(0.342-1.116) | 0.610(0.355-1.004) |
|  | CBPS IPTW - RR (95%CI) | Ref. | 0.575(0.446-0.738)^*^ | 0.543(0.419-0.700)^*^ |
| Non-high risk population | | | | |
|  | No. of events/ no. of patients at risk (%) | 2/230(0.87) | 1/806(0.12) | 2/1503(0.13) |
|  | Crude analysis - RR (95%CI) | Ref. | 0.143(0.007-1.490) | 0.153(0.018-1.275) |
|  | Multivariate^a^ - RR (95%CI) | Ref. | 0.144(0.007-1.500) | 0.150(0.018-1.252) |
|  | CBPS IPTW - RR (95%CI) | Ref. | 0.150(0.038-0.416)^*^ | 0.152(0.039-0.420)^*^ |
| **East Bund population** | | | | |
| Full population | | | | |
|  | No. of events/ no. of patients at risk (%) | 48/2242(2.14) | 9/1561(0.58) | 8/1458(0.55) |
|  | Crude analysis- RR (95%CI) | Ref. | 0.269(0.123-0.522)^*^ | 0.256(0.112-0.511)^*^ |
|  | Multivariate^a^- RR (95%CI) | Ref. | 0.488(0.217-0.990)^*^ | 0.482(0.204-0.993)^*^ |
|  | CBPS IPTW- RR (95%CI) | Ref. | 0.441(0.295-0.648)^*^ | 0.420(0.278-0.622)^*^ |
| High-risk population | | | | |
|  | No. of events/ no. of patients at risk (%) | 47/1405(3.35) | 7/541(1.29) | 4/509(0.79) |
|  | Crude analysis- RR (95%CI) | Ref. | 0.387(0.160-0.801)^*^ | 0.235(0.071-0.577)^*^ |
|  | Multivariate^a^- RR (95%CI) | Ref. | 0.455(0.183-0.978)^*^ | 0.281(0.083-0.717)^*^ |
|  | CBPS IPTW- RR (95%CI) | Ref. | 0.396(0.257-0.594)^*^ | 0.308(0.190-0.478)^*^ |
| Non-high risk population | | | | |
|  | No. of events/ no. of patients at risk (%) | 1/837(0.12) | 2/1020(0.20) | 4/949(0.42) |
|  | Crude analysis - RR (95%CI) | Ref. | 1.641(0.157-35.316) | 3.528(0.522-69.014) |
|  | Multivariate^a^ - RR (95%CI) | Ref. | 1.638(0.157-35.242) | 3.624(0.535-70.957) |
|  | CBPS IPTW - RR (95%CI) | Ref. | 1.513(0.398-6.547) | 3.463(1.162-13.496)^*^ |

CBPS IPTW, covariate-balancing propensity score inverse probability treatment weighting; RR, relative risk.

a: Age, sex and complications were adjusted.

*: statistical significant with p < 0.05.

**Table S12 Effectiveness of inactivated vaccines in reducing viral shedding time in COVID-19 inpatients validated in West Bund population and East Bund population.**

| **Viral shedding time** | **Analysis** | **Incomplete vaccination** | **Full**  **vaccination** | **Booster vaccination** |
| --- | --- | --- | --- | --- |
| **West Bund population** | | | | |
| Full population | | | | |
|  | Median time to recovery (IQR) - days | 14(9-18) | 10(7-13) | 10(7-13) |
|  | Crude analysis- HR(95%CI) | Ref. | 1.789(1.657-1.932)^*^ | 1.818(1.699-1.945)^*^ |
|  | Multivariate^a^- HR (95%CI) | Ref. | 1.281(1.176-1.394)^*^ | 1.267(1.172-1.370)^*^ |
|  | CBPS IPTW- HR (95%CI) | Ref. | 1.254(1.128-1.395)^*^ | 1.205(1.089-1.333)^*^ |
| High-risk population | | | | |
|  | Median time to recovery (IQR) - days | 15(10-19) | 11(7-16) | 11(8-15) |
|  | Crude analysis- HR(95%CI) | Ref. | 1.443(1.293-1.611)^*^ | 1.424(1.296-1.566)^*^ |
|  | Multivariate^a^- HR(95%CI) | Ref. | 1.281(1.143-1.435)^*^ | 1.245(1.126-1.376)^*^ |
|  | CBPS IPTW- HR(95%CI) | Ref. | 1.325(1.164-1.508)^*^ | 1.306(1.179-1.477)^*^ |
| Non-high risk population | | | | |
|  | Median time to recovery (IQR) - days | 10(7-13) | 9(6-12) | 9(7-12) |
|  | Crude analysis- HR(95%CI) | Ref. | 1.221(1.055-1.414)^*^ | 1.223(1.065-1.405)^*^ |
|  | Multivariate^a^- HR(95%CI) | Ref. | 1.244(1.075-1.440)^*^ | 1.254(1.091-1.441)^*^ |
|  | CBPS IPTW- HR(95%CI) | Ref. | 1.216(1.057-1.400)^*^ | 1.230(1.077-1.403)^*^ |
| **East Bund population** | | | | |
| Full population | | | | |
|  | Median time to recovery (IQR) - days | 13(10-16) | 11(8-14) | 11(7-13) |
|  | Crude analysis- HR(95%CI) | Ref. | 1.454(1.364-1.551)^*^ | 1.639(1.534-1.752)^*^ |
|  | Multivariate^a^- HR(95%CI) | Ref. | 1.200(1.122-1.284)^*^ | 1.342(1.252-1.438)^*^ |
|  | CBPS IPTW- HR(95%CI) | Ref. | 1.159(1.063-1.263)^*^ | 1.200(1.067-1.349)^*^ |
| High-risk population | | | | |
|  | Median time to recovery (IQR) - days | 14(10-18) | 12(9-16) | 12(8-15) |
|  | Crude analysis- HR(95%CI) | Ref. | 1.287(1.167-1.420)^*^ | 1.517(1.371-1.679)^*^ |
|  | Multivariate^a^- HR(95%CI) | Ref. | 1.148(1.036-1.271)^*^ | 1.347(1.212-1.497)^*^ |
|  | CBPS IPTW- HR(95%CI) | Ref. | 1.207(1.084-1.343)^*^ | 1.345(1.195-1.513)^*^ |
| Non-high risk population | | | | |
|  | Median time to recovery (IQR) - days | 12(9-14) | 10(7-13) | 10(7-13) |
|  | Crude analysis- HR(95%CI) | Ref. | 1.236(1.128-1.355)^*^ | 1.357(1.236-1.489)^*^ |
|  | Multivariate^a^- HR(95%CI) | Ref. | 1.242(1.134-1.361)^*^ | 1.343(1.223-1.474)^*^ |
|  | CBPS IPTW- HR(95%CI) | Ref. | 1.240(1.136-1.354)^*^ | 1.349(1.233-1.477)^*^ |

IQR, interquartile range; CBPS IPTW, covariate-balancing propensity score inverse probability treatment weighting; HR, hazard ratio.

a: Age, sex and complications were adjusted.

*: statistical significant with p < 0.05.

**Table S13 Peptides identification of RdRp in inactivated vaccines.**

| Protein ID | Sequence | Start position | End position | Length | Charge | m/z | Mass | Mass error [ppm] | Retention time (min) | 1/K0 | CCS | PEP | Score |
| --- | --- | --- | --- | --- | --- | --- | --- | --- | --- | --- | --- | --- | --- |
| P0DTD1 | YSTLQGPPGTGK | 5601 | 5612 | 8 | 2 | 507.793881 | 1013.57321 | -0.33966 | 65.87 | 0.9342255 | 379.2806 | 0.0015716 | 75.915 |
| P0DTD1 | HLIPLMYK | 6073 | 6080 | 18 | 2 | 1009.51184 | 2017.00913 | -0.9296 | 100.77 | 1.218212 | 491.2643 | 0.040961 | 49.097 |
| P0DTD1 | LQSSQAWQPGVAMPNLYK | 6797 | 6814 | 12 | 2 | 603.311677 | 1204.6088 | -1.5715 | 30.301 | 0.899502 | 364.4066 | 0.0019431 | 60.547 |

**Table S14 STROBE Statement.**

|  | |  | | Recommendation | Page No. | |
| --- | --- | --- | --- | --- | --- | --- |
| **Title and abstract** | | 1 | | (*a*) Indicate the study’s design with a commonly used term in the title or the abstract | p. 1 | |
|  |  |  |  | (*b*) Provide in the abstract an informative and balanced summary of what was done and what was found | Submission website | |
| Introduction | | | | | | |
| Background/rationale | | 2 | | Explain the scientific background and rationale for the investigation being reported | p. 1-2 | |
| Objectives | | 3 | | State specific objectives, including any prespecified hypotheses | p. 1-2 | |
| Methods | | | | | | |
| Study design | | 4 | | Present key elements of study design early in the paper | Supplementary material p.4 | |
| Setting | | 5 | | Describe the setting, locations, and relevant dates, including periods of recruitment, exposure, follow-up, and data collection | Supplementary material p.4-6 | |
| Participants | | 6 | | (*a*) *Cohort study*—Give the eligibility criteria, and the sources and methods of selection of participants. Describe methods of follow-up  *Case-control study*—Give the eligibility criteria, and the sources and methods of case ascertainment and control selection. Give the rationale for the choice of cases and controls  *Cross-sectional study*—Give the eligibility criteria, and the sources and methods of selection of participants | Supplementary material p.4-6 | |
|  |  |  |  | (*b*) *Cohort study*—For matched studies, give matching criteria and number of exposed and unexposed  *Case-control study*—For matched studies, give matching criteria and the number of controls per case |  |  |
| Variables | | 7 | | Clearly define all outcomes, exposures, predictors, potential confounders, and effect modifiers. Give diagnostic criteria, if applicable | Supplementary material p.4-5 | |
| Data sources/ measurement | | 8* | | For each variable of interest, give sources of data and details of methods of assessment (measurement). Describe comparability of assessment methods if there is more than one group | Supplementary material p.4-5 | |
| Bias | | 9 | | Describe any efforts to address potential sources of bias | Supplementary material p.4-5 | |
| Study size | | 10 | | Explain how the study size was arrived at | p. 1 | |
| Quantitative variables | 11 | | Explain how quantitative variables were handled in the analyses. If applicable, describe which groupings were chosen and why | | Supplementary material p.7-8 |  |
| Statistical methods | 12 | | (*a*) Describe all statistical methods, including those used to control for confounding | | Supplementary material p.7-8 |  |
|  |  |  | (*b*) Describe any methods used to examine subgroups and interactions | |  |  |
|  |  |  | (*c*) Explain how missing data were addressed | |  |  |
|  |  |  | (*d*) *Cohort study*—If applicable, explain how loss to follow-up was addressed  *Case-control study*—If applicable, explain how matching of cases and controls was addressed  *Cross-sectional study*—If applicable, describe analytical methods taking account of sampling strategy | |  |  |
|  |  |  | (*e*) Describe any sensitivity analyses | |  |  |
| Participants | 13* | | (a) Report numbers of individuals at each stage of study—eg numbers potentially eligible, examined for eligibility, confirmed eligible, included in the study, completing follow-up, and analysed | | p. 1 |  |
|  |  |  | (b) Give reasons for non-participation at each stage | |  |  |
|  |  |  | (c) Consider use of a flow diagram | |  |  |
| Descriptive data | 14* | | (a) Give characteristics of study participants (eg demographic, clinical, social) and information on exposures and potential confounders | | Supplementary material p.4-5 |  |
|  |  |  | (b) Indicate number of participants with missing data for each variable of interest | | N/A |  |
|  |  |  | (c) *Cohort study*—Summarise follow-up time (eg, average and total amount) | | Supplementary material p.4-5 |  |
| Outcome data | 15* | | *Cohort study*—Report numbers of outcome events or summary measures over time | | p.1-4 |  |
|  |  |  | *Case-control study—*Report numbers in each exposure category, or summary measures of exposure | |  |  |
|  |  |  | *Cross-sectional study—*Report numbers of outcome events or summary measures | |  |  |
| Main results | 16 | | (*a*) Give unadjusted estimates and, if applicable, confounder-adjusted estimates and their precision (eg, 95% confidence interval). Make clear which confounders were adjusted for and why they were included | | p. 1-4 |  |
|  |  |  | (*b*) Report category boundaries when continuous variables were categorized | |  |  |
|  |  |  | (*c*) If relevant, consider translating estimates of relative risk into absolute risk for a meaningful time period | |  |  |
| Other analyses | 17 | | Report other analyses done—eg analyses of subgroups and interactions, and sensitivity analyses | | p. 1-4 |  |
| Key results | 18 | | Summarise key results with reference to study objectives | | p. 1-4 |  |
| Limitations | | 19 | Discuss limitations of the study, taking into account sources of potential bias or imprecision. Discuss both direction and magnitude of any potential bias | | p. 4 |  |
| Interpretation | | 20 | Give a cautious overall interpretation of results considering objectives, limitations, multiplicity of analyses, results from similar studies, and other relevant evidence | | p. 1-4 |  |
| Generalisability | | 21 | Discuss the generalisability (external validity) of the study results | | p. 4-5 |  |
| **Other information** | |  |  | |  |  |
| Funding | | 22 | Give the source of funding and the role of the funders for the present study and, if applicable, for the original study on which the present article is based | | p. 5 |  |

**Table S15 Definitions of comorbidities.**

| **Comorbidities** | **Specific diseases** |
| --- | --- |
| Cardiovascular diseases | Hypertension, coronary heart disease, atrial fibrillation, old myocardial infarction, cardiac insufficiency, history of venous thromboembolism, and pacemaker implant status |
| Cerebrovascular and neurologic diseases | Cerebral infarction, cerebral haemorrhage, and Parkinson's disease |
| Hepatic diseases | Liver cirrhosis and liver insufficiency |
| Pulmonary complications | Chronic obstructive pulmonary disease, pulmonary fibrosis, and asthma |
| Chronic kidney diseases | Chronic renal insufficiency and treatment of long-term renal replacement therapy |
| Metabolic diseases | Diabetes and hyperlipidemia |
| Tumors | Haematological tumor and solid malignancies |
| Immunodeficiency diseases | Primary immunodeficiency, HIV infection, system use of hormonal drugs, long-term use of immunosuppressive drugs, autoimmune disease, solid organ transportation, and stem cell transplant |

**Table S16 Clinical severity classification.**

| **Clinical severity classification** | **Criteria** |
| --- | --- |
| Asymptomatic infection | Confirmed SARS-CoV-2 infection cases without clinical symptoms |
| Mild infection | Infections who had mild symptoms without signs of pneumonia on chest imaging |
| Moderate infection | Infections who had signs of pneumonia on radiologic assessment |
| Severe infection | Infections with one of the following conditions:  1) Respiration rate ≥ 30/min  2) SpO_2_ ≤ 93% in resting state  3) arterial partial pressure of oxygen/fraction of inspired oxygen ≤ 300 mmHg  4) cases with progressive clinical aggravation or chest imaging that shows lesion progression > 50% within 24-48 hours |
| Critical infection | Infections with one of the following conditions:  1) respiratory failure requiring mechanical ventilation  2) shock  3) a combination of other organ failures requiring ICU care |

**Table S17 Definitions of outcomes.**

| **Outcome** | **Definition** |
| --- | --- |
| Pneumonia | Pneumonia was diagnosed by experienced doctors in the COVID-19 designated hospitals according to radiologic assessment. And the radiologic data were re-diagnosed by another group of professional doctors for confirmation in this analysis. |
| Severe outcomes | 1. Inpatients treated with high-flow oxygen, tracheal intubation, tracheotomy, blood purification, Extracorporeal Membrane Oxygenation (ECMO), mechanical ventilation, and vasoactive drugs or who had a combination of severe organ failures, and who had instable hemodynamic were often transferred to ICU. 2. Inpatients died of the disease progression during hospitalization. |
| Disease progression | Inpatients whose diagnosis classification at discharge was severer than the diagnosis at admission were considered as developing disease progression, such as the one who was diagnosed with asymptomatic infection at admission and moderate infection at discharge. And severe/critical infections at admission were excluded. |
| Viral shedding time | The time duration from the first date of a positive nucleic acid test to the first date of two consecutive negative tests with an interval of over 24 hours. The cycle threshold value which was large than 35 in at least ORF1ab and N gene was defined as negative result. |
